# Infrared Spectroscopy Elucidates the Inhibitor Binding Sites in a Metal‐Dependent Formate Dehydrogenase

**DOI:** 10.1002/chem.202201091

**Published:** 2022-08-03

**Authors:** Konstantin Laun, Benjamin R. Duffus, Stefan Wahlefeld, Sagie Katz, Dennis Belger, Peter Hildebrandt, Maria Andrea Mroginski, Silke Leimkühler, Ingo Zebger

**Affiliations:** ^1^ Institut für Chemie Max-Volmer-Laboratorium für Biophysikalische Chemie PC14 Technische Universität Berlin Strasse des 17. Juni 135 10623 Berlin Germany; ^2^ Institut für Biochemie und Biologie Molekulare Enzymologie Universität Potsdam Karl-Liebknecht-Strasse 24–25 14476 Potsdam Germany; ^3^ Institut für Technische Biokatalyse Technische Universität Hamburg Denickestr. 15 21073 Hamburg Germany

**Keywords:** CO_2_ reduction, DFT, formate oxidation, inhibition kinetics, IR spectroscopy, molybdoenzyme

## Abstract

Biological carbon dioxide (CO_2_) reduction is an important step by which organisms form valuable energy‐richer molecules required for further metabolic processes. The Mo‐dependent formate dehydrogenase (FDH) from *Rhodobacter capsulatus* catalyzes reversible formate oxidation to CO_2_ at a bis‐molybdopterin guanine dinucleotide (bis‐MGD) cofactor. To elucidate potential substrate binding sites relevant for the mechanism, we studied herein the interaction with the inhibitory molecules azide and cyanate, which are isoelectronic to CO_2_ and charged as formate. We employed infrared (IR) spectroscopy in combination with density functional theory (DFT) and inhibition kinetics. One distinct inhibitory molecule was found to bind to either a non‐competitive or a competitive binding site in the secondary coordination sphere of the active site. Site‐directed mutagenesis of key amino acid residues in the vicinity of the bis‐MGD cofactor revealed changes in both non‐competitive and competitive binding, whereby the inhibitor is in case of the latter interaction presumably bound between the cofactor and the adjacent Arg587.

## Graphical abstract

**Metal‐dependent formate** dehydrogenases (FDH) are capable of oxidizing formate to CO_2_ reversibly. Here, we compared IR spectroscopic data with DFT calculations and inhibition kinetics to study the putative binding mode of CO_2_/formate at the bis‐MGD cofactor with the help of isoelectronic and charged product analogues. Experimental and theoretical data exclude a direct binding of the inhibitor molecules to the bis‐MGD.

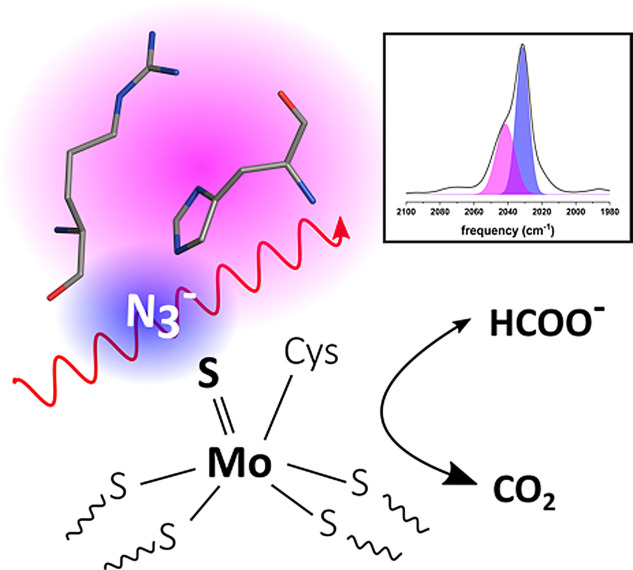

## Introduction

In light of the impact of carbon dioxide on the climate, catalysts capable of converting CO_2_ into fuels are of great interest.^[1]^ Metal‐containing formate dehydrogenases (FDH) perform the reversible oxidation of formate to yield CO_2_, one proton, and two electrons under physiological conditions.^[2]^ Thus, these enzymes are also candidates for potential biotechnological applications and have been extensively studied in the field of (photo)electrochemical generation of carbon‐based fuels with regard to bio‐fuel cells and photoelectrochemical tandem devices as well as for the regeneration of natural and non‐natural redox cofactors.^[3]^ The herein studied FDH from *Rhodobacter capsulatus* (*Rc*FDH) consists of a (αβγδ)_2_ dimer of heterotetramers harboring a bis‐molybdopterin guanine dinucleotide (bis‐MGD) cofactor, in addition to five [4Fe‐4S] clusters, two [2Fe‐2S] clusters, and a flavin mononucleotide (FMN) prosthetic group.^[4]^ Nicotinamide adenine dinucleotide (NAD^+^) is the physiological electron acceptor.[^2a^, ^4^, ^5^] In its resting state, the Mo^VI^ ion is coordinated by four sulfurs from two MGD dithiolenes, a sulfido ligand and a cysteine (Cys386) ligand from the protein backbone.[^2a^, ^4^] Upon two electron reduction, the Mo^IV^ ion is proposed to be pentacoordinated due to the displacement of Cys386.^[6]^

**Figure 1:**
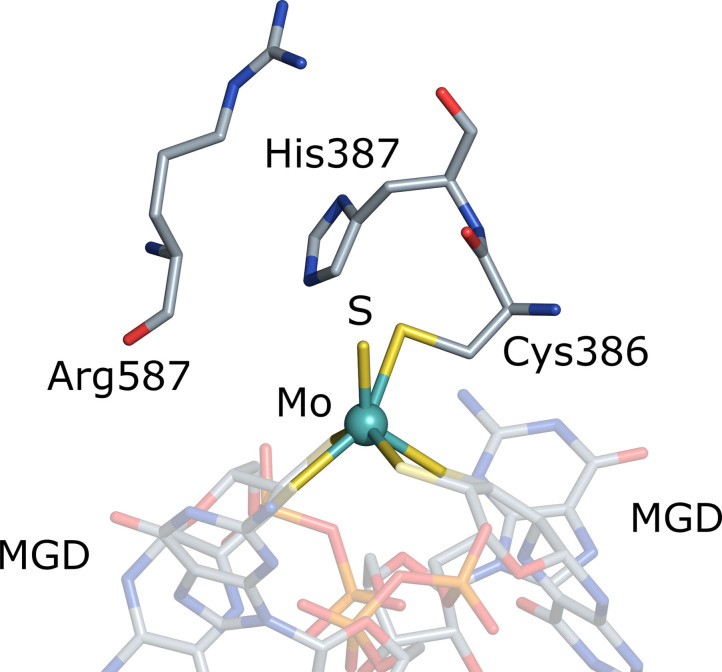
Active site and ligand environment of the bis‐MGD cofactor derived from the recently published corresponding cryo‐EM structure of the “as isolated” form of the formate dehydrogenase from *Rhodobacter capsulatus* (PDB entry 6TGA).^[4]^ Here, the hexacoordinated molybdenum ion is ligated by a sulfido group, Cys386 and four sulfur atoms belonging to the dithiolenes from two MGDs. The highlighted conserved amino acids Arg587 and His387 play a crucial role for inhibitor binding in the second coordination sphere.

Furthermore, important amino acid residues in the second coordination sphere, present in all metal‐containing FDH enzymes, include a highly conserved histidine (His387) and an arginine (Arg587). Both have been proposed to ensure optimal substrate binding to and proton transfer away from the active site (Figure 1).[^6a^, ^7^] The exact catalytic mechanism is currently still under debate.[^6b^, ^7^] In previous studies, azide and cyanate (N_3_^−^ and OCN^−^), both isoelectronic and isostructural molecules to CO_2_ ^[8]^ but charged as formate, have been considered as analogues to putative intermediate states of the catalytic cycle.^[9]^ Moreover, they have been identified as inhibitors of FDH enzymes.^[10]^ In addition, azide is also used as a protectant during the purification of such proteins to prevent oxidative inactivation of the enzyme caused by the replacement of the essential sulfido ligand by an oxo ligand, as recently shown by EXAFS spectroscopy.^[12]^ However, the exact inhibitor binding site remained elusive. As basis for deeper mechanistic studies, such inhibitors were chosen to elucidate potential substrate binding sites in the bis‐MGD‐containing FDH−H from *Escherichia coli* by employing in depth protein film electrochemical investigations, that suggested an oxidation state‐dependent competitive inhibition by various compounds including azide and cyanate, which act supposedly as π‐electron donors to the Mo^VI^ ion in a coordinate bond.^[7c]^ However, no structural evidence could be provided by this technique.

In the present study, infrared (IR) spectroscopy was employed in combination with density functional theory (DFT) and inhibition kinetic studies to elucidate the specific interaction of such inhibitory CO_2_ analogues with *Rc*FDH. The negative charge and hence the large transition dipole moment of the inhibitory anions causes high extinction coefficients of the antisymmetric stretching modes, which are exceptionally well‐suited for IR measurements as they are located in a spectral region free of any protein or water absorptions.^[12]^ Moreover, as these particular vibrational reporters are highly sensitive to changes in the electronic structure in their molecular environment, they can be used as marker bands.^[13]^ By characterizing the binding of these anions and their selected isotopologues to the *Rc*FDH and associated variants, potential interaction sites at the bis‐MGD and in its secondary coordination sphere could be identified. In addition, we have investigated the azide inhibition of the inactive oxo‐ligated bis‐MGD that was produced in absence of its maturation precursor, *Rc*FDH^ΔFdsC^.^[14]^ In order to elucidate the modality of the bis‐MGD dependent binding, the calculated spectra derived from conceivable DFT models were compared to the experimentally obtained vibrational bands of protein‐bound azide and cyanate.

## Results and Discussion

**Figure 2:**
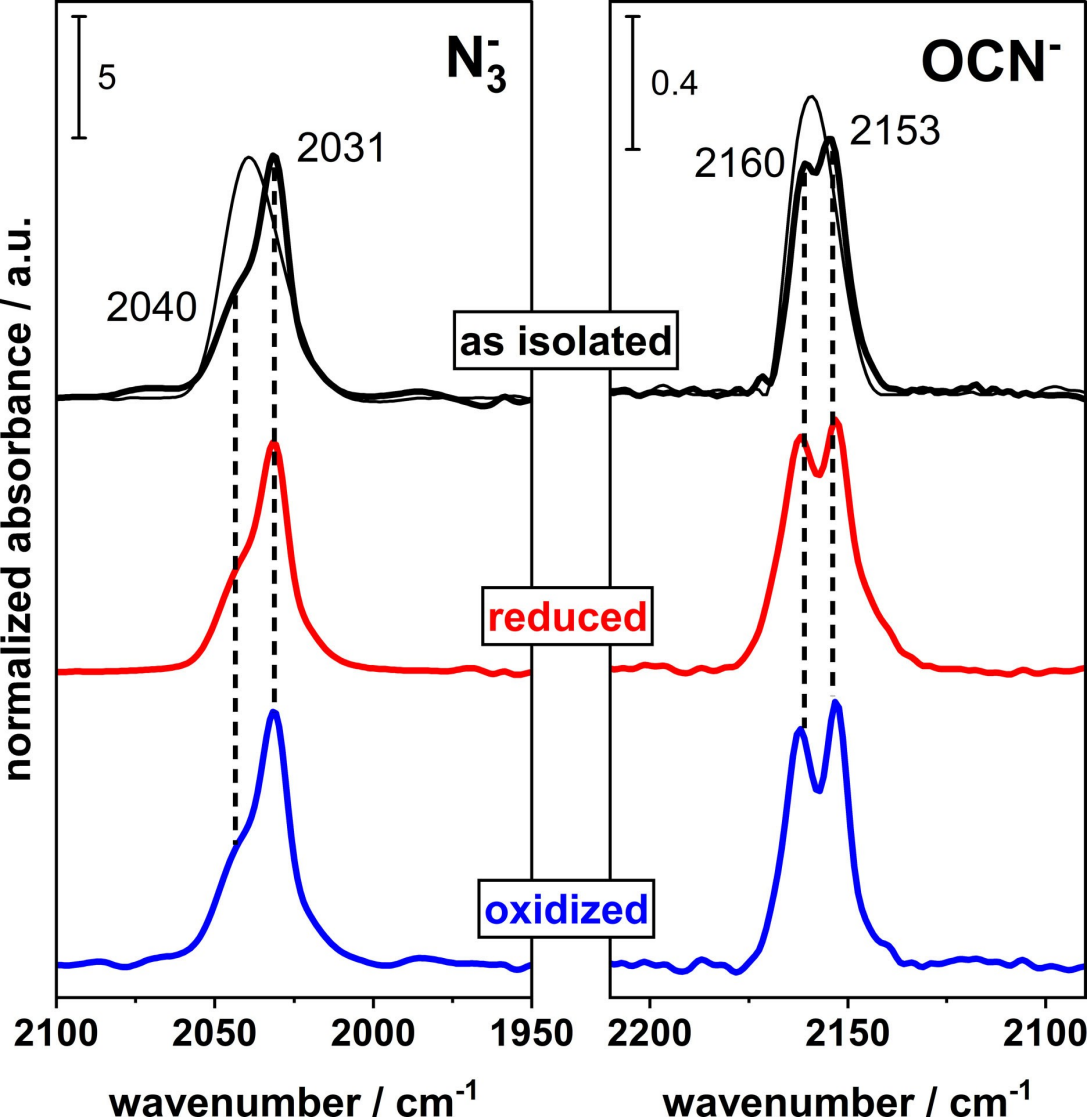
Normalized IR spectra of *Rc*FDH^WT^ samples in different redox states treated with 10 mM azide (left panel) and 10 mM cyanate (right panel) after subtraction of the spectral contributions from the free inhibitors in solution and baseline correction. Further details are given in the Figure S1. The binding of azide to the *Rc*FDH^WT^ gives rise to two major bands at 2031 and 2040 cm^−1^. Cyanate incubation results in the formation of two bands at 2153 and 2160 cm^−1^. The obtained spectra of inhibited “as isolated” samples are plotted in black. Spectra of the apoenzyme (*Rc*FDH^Δbis−MGD^) incubated with the respective inhibitors are displayed in grey, exhibiting a broader absorption at higher frequencies. The incorporation of the bis‐MGD cofactor in the protein is reflected the presence of a second band at slightly lower wavenumbers (Δ*ν* ≈7–9 cm^−1^). No change in the respective band positions was observed upon reduction or oxidation of the protein sample with 10 mM sodium formate or thionine (depicted as red and blue traces), respectively.

Following the procedure described in Figure S1, all baseline corrected and to amide II band normalized IR spectra shown here, were obtained from *Rc*FDH proteins incubated with 10‐fold excess inhibitor in which contributions of free inhibitors in solution were subtracted. The spectra of the as isolated protein sample treated with azide and cyanate (Figure 2 top traces) exhibit each two major absorption bands. Both bands are red shifted compared to those of the free inhibitors in water (Figure S1). The first band, found at 2040 cm^−1^ for azide‐ and at 2160 cm^−1^ for cyanate‐treated samples, was also observed in the apoprotein (*Rc*FDH^Δbis−MGD^) (Figure 2, gray traces).

The small frequency shift (Δ*ν* ≈8 cm^−1^) and the detected band shape suggest a conformation of the azide, which is more similar to that of free azide in water (Figure S1). The second distinct band, observed at 2031 cm^−1^ for azide, and 2153 cm^−1^ for cyanate treatment, could only be detected in the bis‐MGD‐containing enzyme (Figure 2). Thus, these bands could be assigned to a specific interaction site of an inhibitor molecule with the bis‐MGD cofactor. Moreover, all bands were not observed in corresponding control experiments, comparing both azide binding to the diaphorase subcomplex FdsGB that lacks the α‐subunit harboring the bis‐MGD cofactor, and bovine serum albumin (BSA). Based on the spectral results of these control experiments, we can exclude unspecific interactions of azide with globular proteins in general and in particular with other subunits of the *Rc*FDH (Figure S2).

To elucidate the specific role of the different binding sites, the concentrations of both types of bound azide molecules were estimated from their band intensities (*A*_int_) within a semi‐quantitative evaluation (Figures S3 and S4). They were found to be in a similar order of magnitude as the protein concentration, yielding in sum values about 0.5 anions per *Rc*FDH molecule. As this observation was consistent for all azide‐incubated samples, it rather points to a modified extinction coefficient due to the interaction with the protein environment^[12a]^ and thus an underestimation of the amount of bound inhibitor. Previous studies have reported that azide acts as a protectant in a stoichiometric fashion.[^7a^, ^11b^] Therefore, it is appropriate to assume a stochiometric 1 : 1 ratio between inhibitor and protein molecules. The bis‐MGD cofactor (Mo) loading of approximately 50 % was also considered.

Moreover, this result indicates that in presence of the bis‐MGD cofactor, the azide molecule from the interaction site, related to the absorption at 2040 cm^−1^, forms the binding site indicated by the band at 2031 cm^−1^. This finding is supported by the fact that the overall integrated absorbance *A*_int_ of both bands (*A*_int_(2031+2040 cm^−1^)) in the *Rc*FDH^WT^ sample is equivalent to the total integral *A*_int_ of the band at 2040 cm^−1^ in the apoenzyme, *Rc*FDH^Δbis−MGD^. The calculated difference spectrum, in which spectral contribution of the apoenzyme with respect to the Mo loading are subtracted, shows exclusively the remaining bis‐MGD dependent band centered at 2031 cm^−1^ (see Figure S4). This observation suggests that the two observed bands represent two populations of potential binding sites within the sample (apo‐ and holoenzyme), to which in total only one inhibitory molecule can bind.

**Figure 3:**
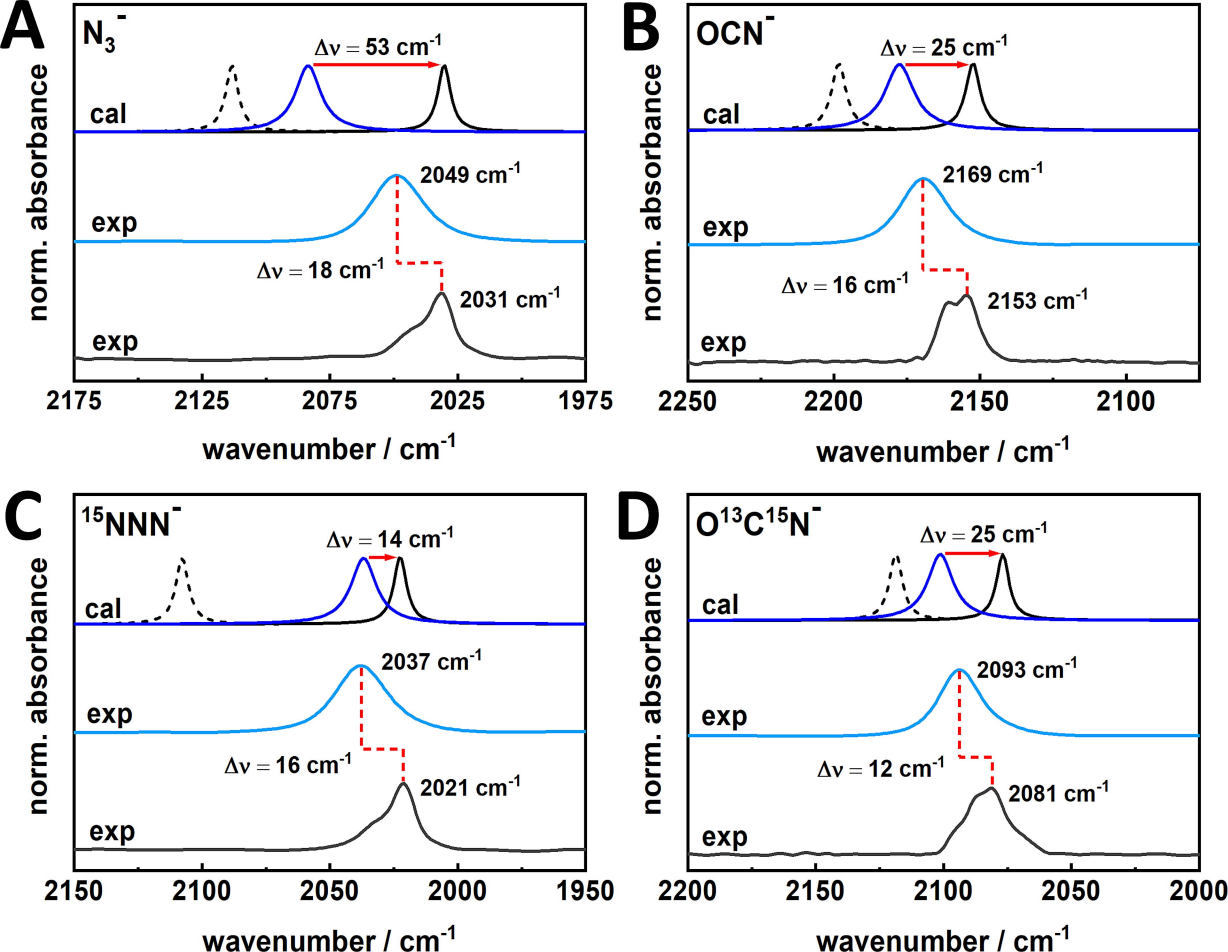
Comparison of DFT‐predicted (cal) and experimental (exp) IR absorption bands related to the OCN^−^, NNN^−^ inhibitors and their isotopologues. The corresponding asymmetric stretching frequencies of the inhibitors were calculated for a covalent Mo−N/^15^N/O bond (bound, dashed back lines) and a non‐covalent, pure electrostatic interaction near the bis‐MGD cofactor (unbound, solid black lines) and compared to the corresponding band positions of free inhibitors in water (solid dark blue line). A non‐covalent interaction would thus result in a red shift of the inhibitor bands as depicted by red arrows. The experimental results of the *Rc*FDH^WT^ incubated with the respective inhibitors exhibit two red shifted absorption bands (black solid trace, exp) relative to the inhibitor bands in water (blue solid trace, exp) for A) NNN^−^ and B) OCN^−^. The same results were obtained by using isotopically labelled azide and thiocyanate, again in good agreement with C, D) the DFT predictions. Asterisk represents non‐substracted free O^13^C^15^N^−^ in H_2_O (D).

Complementary DFT calculations were performed to clarify the modality of the bis‐MGD dependent binding. For this purpose, DFT calculations on structural models comprising a bis‐MGD cofactor (Mo^VI^), the ligated Cys386, and an additionally incorporated inhibitor anion were conducted (Figure S5). The corresponding Mo coordination geometry was derived from the oxidized structure of the related bis‐MGD of the nitrate‐inducible formate dehydrogenase (FDH−N) from *E. coli*.^[15]^ Due to the inherent error of the spectra calculation, the absolute positions of IR bands do not reflect experimental data accurately. However, the calculated trends related to changes of the structure or the electronic environment can be directly correlated with the corresponding experimental data. The calculations refer to two possible inhibitor interaction scenarios. In one case, the Mo ion remained hexacoordinated (i. e., Cys386 is still bound) in a Mo^VI^ oxidation state and the respective inhibitor was kept in an energetically favored position near the Mo^VI^ ion but excluding a covalent Mo−N bond (see Figure S6 and Table 1 in the Supporting Information). The second scenario was based on the inhibitor binding according to a proposed catalytic mechanism where the substrate binds at the metal center.^[7c]^ Here, the Mo ion resided in a reduced Mo^IV^ state and the thiolate bond to Cys386 was replaced by a covalent bond to the respective azide or cyanate molecule (Mo^IV^‐N/O). DFT calculations were also performed for the corresponding interactions with isotopically labeled azide (^15^N=N=N^−^) and cyanate (O=^13^C=^15^N^−^) for a proper comparison with the experimental spectra (see Figures 3, S6 and S7). The shifts of the two adjacently and partially overlapping experimentally derived IR bands relative to the band of azide in solution are in good agreement, both in terms of direction and magnitude, with an interaction site that does not involve direct coordination to the Mo ion. This is also in line with former investigations on azide binding in Cu,Zn superoxide dismutase as discussed in more detail previously^[16]^ and supported by the fact that no significant band shifts were observed upon oxidation or reduction of *Rc*FDH samples with an excess of thionine and formate, respectively (Figure 2, middle and bottom traces).

**Table 1:** Formate oxidation constants and inhibition kinetic data^[a]^ for *R. capsulatus* FDH.

		FDH^WT^	FDH^WT^	FDH^H387M^	FDH^H387M^	FDH^R587K^	FDH^R587K^
		DCPIP	NAD^+^	DCPIP	NAD^+^	DCPIP	NAD^+^
	*K*_formate_ [μM]	151±6		29275±1647		401750±12884	
	*k*_cat_ [min^−1^]	1804±44		956±41		812±13	
N_3_^−^	*K*_i_ [μM]	47±3	48±3	517±37	476±14	7384±458	4840±226
	*αK*_i_ [μM]	1378±16	1372±17	5656±84	6391±38	–	–
OCN^−^	*K*_i_ [μM]	783±48	780±81	7841±276	8079±265	8137±418	5571±613
	*αK*_i_ [μM]	11755±88	12451±131	27497±340	30369±386	–	–

[a] Further information are given in the Supporting Information.

Enzyme reduction is illustrated in the corresponding UV‐visible absorption spectra by the respective intensity loss of bands characteristic for Fe−S clusters and FMN (Figure S8). In addition, previous XAS and EPR spectroscopic studies have proven that such treatments change the oxidation state of the Mo ion to Mo^V^ or Mo^IV^.^[11]^ So far, our DFT and IR data suggest that the bis‐MGD‐independent band at higher frequencies represents an inhibitor species solely in the *Rc*FDH^Δbis−MGD^ (apoenzyme), whereas the lower frequency band appears to refer to a specific interaction site in the *Rc*FDH^WT^ (holoenzyme). Notably, the observed binding‐site‐specific bands remain independent from the cofactor's oxidation state, indicating that none of the inhibitors coordinate directly the Mo ion. This has been also recently proposed within a combined quantum mechanical and molecular mechanic calculations.^[17]^

**Figure 4:**
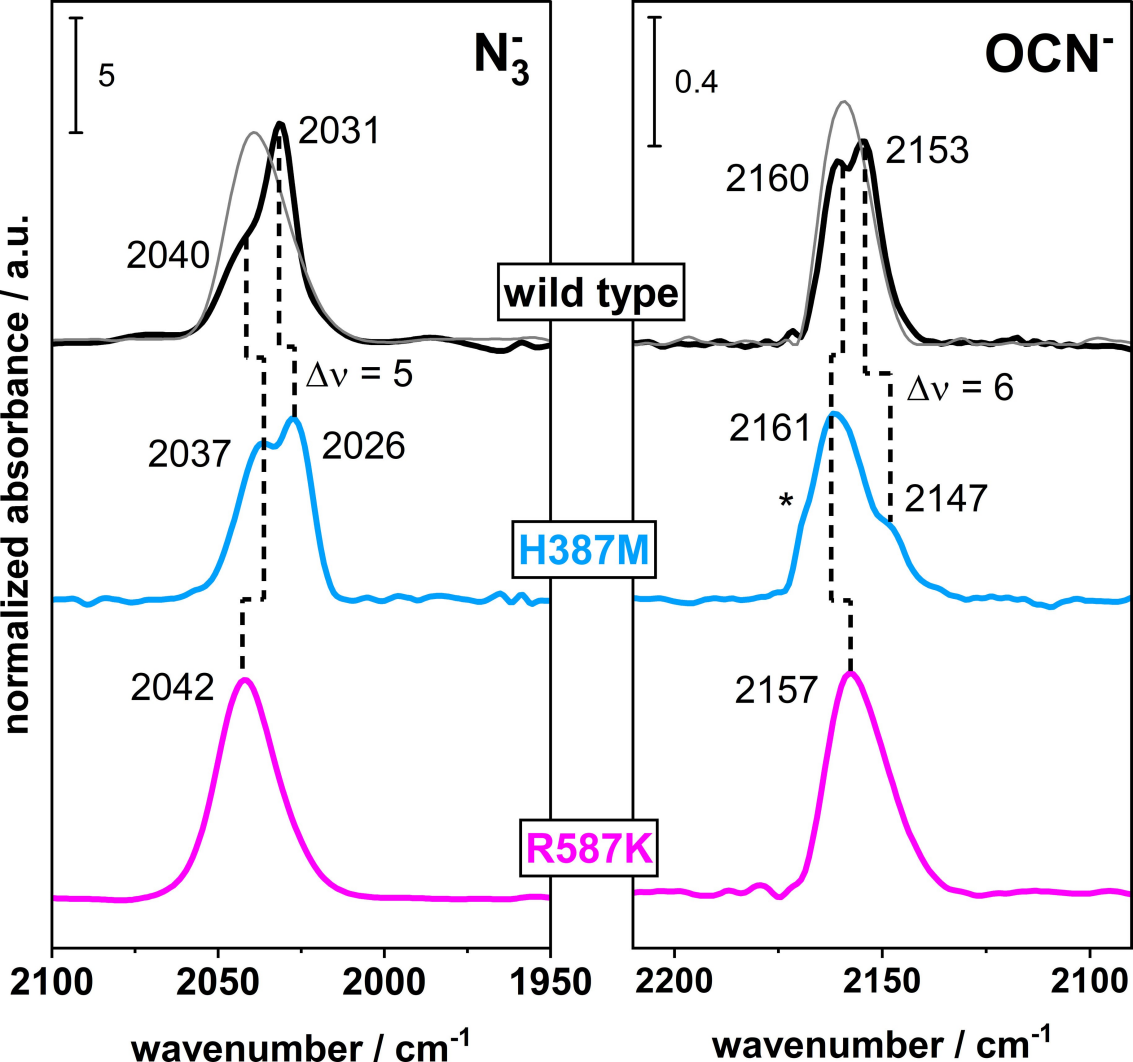
Normalized IR spectra of inhibited *Rc*FDH variants. Azide (left panel) and cyanate (right panel) inhibition was performed with *Rc*FDH^WT^, *Rc*FDH^H387M^ and *Rc*FDH^R587K^. Spectrum displayed in grey was obtained from apoprotein, *Rc*FDH^Δbis−MGD^, and is assigned to the non‐competitive inhibition interaction site. A small intensity loss as well as a shift (Δ*ν*=5 cm^−1^) is detected for the band assigned to the bis‐MGD‐dependent inhibition site in *Rc*FDH^H387M^ samples treated with azide. A more significant loss of intensity and a shift of Δ*ν*=6 cm^−1^ is observed for cyanate inhibition. When Arg587 is replaced (*Rc*FDH^R587K^ and *Rc*FDH^H387M/R587T^), an absorption assigned to an apparent competitive interaction of the respective inhibitor can be detected, due to changes in competitive inhibitor binding relative to *Rc*FDH^WT^. Further details related to *Rc*FDH^H387M/R587T^ are given in the Figure S9. Spectra were measured in 100 mM Tris‐HCl pH 9.0 (10 °C) and 10 mM of the respective inhibitor. The asterisk labels residual spectral contribution of free cyanate in water.

Moreover, previous studies have suggested that the conserved His387 and Arg587 residues, located in the second coordination sphere of bis‐MGD, are involved in both substrate binding and stabilization in the protein scaffold.[^7a^, ^9^, ^11a^] To evaluate the role of these key residues for binding of inhibitors, two active protein variants *Rc*FDH^H387M^ and *Rc*FDH^R587K^ (see Figures 4 and S9) were prepared and biochemically characterized including the respective inhibition kinetics (Table 1).

In catalytic assays for formate oxidation, using NAD^+^ as an electron acceptor to interact with the FMN or DCPIP (2,6‐dichlorophenolindophenol) as an electron acceptor at the bis‐MGD‐site, *k*_cat_ values of about 1800 min^‐1^ were obtained for *Rc*FDH^WT^, while lowered *k*_cat_ values were observed for the *Rc*FDH^H387M^ and *Rc*FDH^R587K^ variants.^[11a]^ For the latter variant, a large *K*_M_ (formate) value was obtained, displaying a significantly lower binding affinity. While *Rc*FDH^WT^ and *Rc*FDH^H387M^ revealed a mixed‐type inhibition for azide and cyanate, the amino acid exchange in the *Rc*FDH^R587K^ variant resulted in a strictly competitive inhibition, which underlines the importance of Arg587 for both substrate and competitive inhibitor binding.

Notably, significant differences were observed for the inhibition constants of azide and cyanate. In comparison to *K*_i_ (azide) of *Rc*FDH^WT^, the *K*_i_ (azide) of the active site variants *Rc*FDH^H387M^ and *Rc*FDH^R587K^ increased by a factor of ∼10 and 120, respectively (Table 1). By comparison, the *K*_i_ (cyanate) of the *Rc*FDH^WT^ has increased by a factor of ∼15 relative to *K*_i_ (azide). This suggests a stronger influence of these amino acids on the binding of azide compared to cyanate. Furthermore, azide is also a more efficient inhibitor as reflected by the lowered *K*_i_ and *αK*_i_ values.

Additionally, no influence on the *K*_i_ values was observed in experiments using DCPIP (2,6‐dichlorophenolindophenol) as electron acceptor at the bis‐MGD, confirming that both forms of inhibition rather occur near the active site instead of being related to an interruption of the intramolecular electron transfer.^[2b]^

Complementary to the biochemical assays, IR spectra of the azide‐ and cyanate‐treated variants were recorded (Figure 4). While the IR characteristic band doublet was observed in the spectrum of the *Rc*FDH^H387M^ variant for each of the two inhibitors, albeit slightly shifted compared to the WT protein, only the higher frequency component, as found for the *Rc*FDH^▵bis−MGD^, was detected in the spectrum of the *Rc*FDH^R587K^ variant at 2042 and 2157 cm^−1^ for azide and cyanate, respectively. These data suggest that the binding site of the inhibitor, giving rise to the lower frequency IR bands at 2031 and 2153 cm^−1^ for azide and cyanate, respectively, is localized between the bis‐MGD cofactor, presumably the sulfido ligand, and the Arg587 in a more defined manner. This binding site would correspond to the competitive inhibition mode component as derived from the kinetic experiments of *Rc*FDH^WT^ and *Rc*FDH^H387M^.

**Figure 5:**
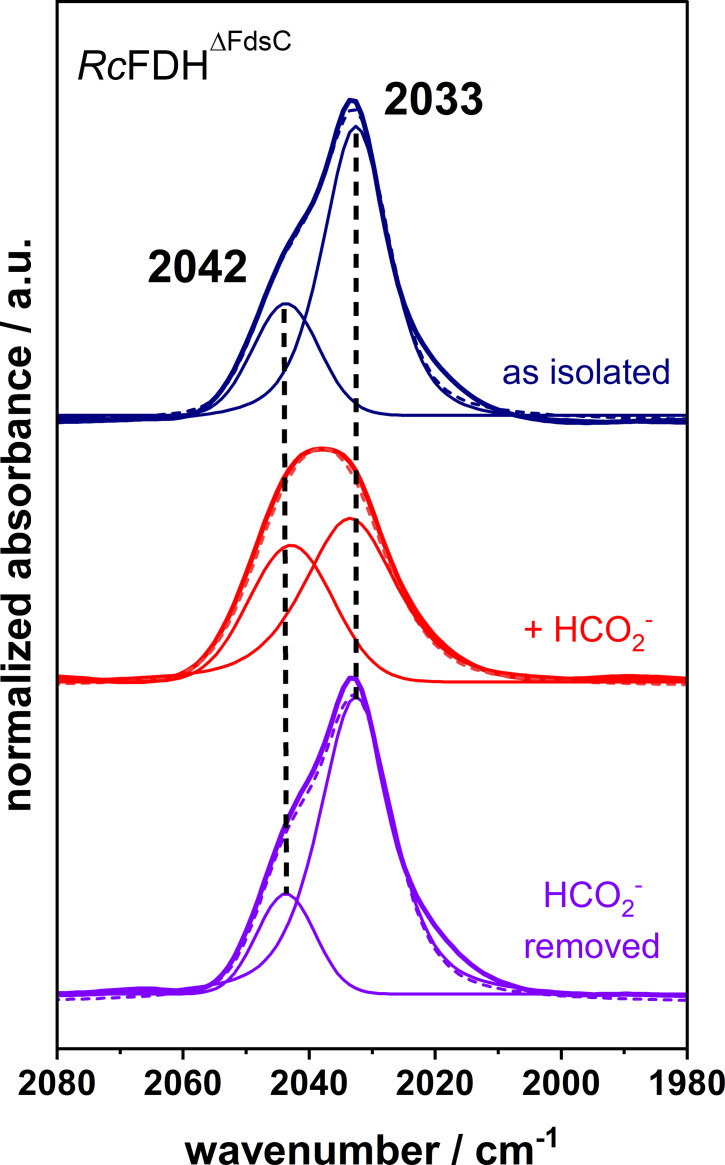
Normalized IR spectra of azide inhibited *Rc*FDH^ΔFdsC^ variants. The inactive oxo‐ligated bis‐MGD cofactor embedded in *Rc*FDH was characterized in the “as isolated” form (dark blue), in presence of formate (red) and after removal of formate (purple). Solid lines represent the fits of individual band contributions, whereas the dashed line display the respective experimental spectrum. Similar to *Rc*FDH^WT^, two main bands can be deconvoluted. The absorption of the competitively bound azide at 2033 cm^−1^ (dark blue) is shifted towards slightly higher frequencies compared to *Rc*FDH^WT^. This could be explained by the exchange of the sulfido ligand, which was previously identified as putative interaction site of the competitively inhibiting azide, to an oxo ligand. The competitively inhibiting azide in this position was displaced in the presence of formate, thereby, forming the second observed, apo‐protein‐like bound azide at about 2042 cm^‐1^ (red). Upon removal of the substrate formate, the azide flips back from the non‐competitive to the competitive binding site (purple).

The interaction site at the bis‐MGD was further investigated by comparing an inactive oxo‐ligated bis‐MGD embedded in *Rc*FDH^ΔFdsC^ with the active *Rc*FDH^WT^ enzyme (Figures 5 and S10). In the presence of the oxo ligand (Mo=O), the bis‐MGD dependent band related to competitive binding azide, shifts from 2031 cm^−1^ to 2033 cm^−1^, indicating the interaction of the azide molecule with the oxo‐ligated Mo ion. The addition of formate leads to the displacement of azide from the binding site reflected by the replacement of the lower frequency IR band at 2033 cm^−1^ with a band at about 2042 cm^−1^ (Figure 5). This translocation of the inhibitor was found to be reversible, which is consistent with the observed mixed‐type inhibition observed in *Rc*FDH^WT^ . These findings imply that azide can remain in the active site pocket under non‐turnover conditions and also in the presence of the substrate which would explain the non‐competitive component of azide inhibition, probably by interrupting the rapid flow and alignment of substrate and product to and from the active site, respectively.

Nevertheless, the exact location of the non‐competitive binding site of the inhibitor could not be conclusively inferred from our data. It is reasonable to assume that this site is located in the proximity of the competitive binding site but rather exhibits differences in its conformation, that is more similar to those of free azide in water (*ν*_freeN3_=2049 cm^−1^) (for further explanation, see Figure S4). Presumably, it is bound to the protein in a manner, which provides a higher degree of translational freedom. This assumption would explain the small spectral shifts in the inhibitor‐treated *Rc*FDH^WT^, *Rc*FDH^R587K^ and *Rc*FDH^H387M^ holoenzyme variants (Figure 4). The respective apoenzyme variants without the bis‐MGD cofactor confirm these small spectral shifts of the stretching vibrations of the azide molecule at higher frequencies (Figure S11). Notably, also the non‐competitive binding site seems to be relevant for catalysis. The azide molecule remains in the pocket during catalysis and, hence, would contribute to a mixed‐type inhibition for *Rc*FDH^WT^ and *Rc*FDH^H387M^ as well as an apparent competitive inhibition for *Rc*FDH^R587K^.

## Conclusion

By means of a combined approach including inhibition kinetics, site directed mutagenesis, DFT calculations and IR spectroscopy, we have elucidated the binding motifs of inhibitory azide and cyanate anions in the Mo‐dependent formate dehydrogenase. It was found, that, independent of the oxidation state of the Mo ion, one inhibitor can electrostatically interact with two different binding sites in the secondary coordination sphere of the bis‐MGD Mo center. These interaction sites were correlated with the inhibition modes suggested by enzyme kinetic assays and were assigned to a competitive and a non‐competitive binding site, whereby the latter is presumably located more remote from the bis‐MGD cofactor.

Since the inhibitors are isoelectronic and isostructural with CO_2_, but also charged as formate, they may serve as models for substrate or product binding to FDH. Hereby, the competitive binding site could contribute to a stabilization of formate/CO_2_ molecules between the crucial Arg587 and the sulfido ligand of the bis‐MGD cofactor during catalysis. This particular binding motif might also explain the increased oxygen stability of azide‐ or cyanate‐inhibited enzymes by sterically shielding the sulfido ligand against oxidative damage. In addition, also the adjacent His387 plays a supportive role for inhibitor binding. The importance of both amino acid residues in the binding of formate/CO_2_ has been discussed in a very recent computational study on the active site structure of *Ec*FDH−N.[^6a^, ^11b^] Moreover, the specific interaction site was confirmed by an experiment in which the inactive, oxo‐ligated bis‐MGD in *Rc*FDH^ΔFdsC^ was incubated with azide, exhibiting clearly its displacement from the competitive binding site accompanied by a migration towards the non‐competitive binding site. However, a direct binding of CO_2_ or formate to the Mo ion during catalysis cannot be entirely excluded from our static studies, but it seems to be unfavored.[^9^, ^17^] In summary, the present study provides the basis for future mechanistic investigations of Mo/W‐dependent formate dehydrogenases and other metalloenzymes, which may be deepened by (ultrafast) time‐resolved, non‐linear IR methods targeting at the transitional state as demonstrated by the work of Cheatum and co‐workers.^[18]^ Such results could potentially provide valuable information for the optimization of biotechnological applications using for example electro‐ or photocatalytic reduction of formate in bio‐fuel cells and photoelectrochemical tandem devices,[^3a^, ^3b^] as well as for future blueprints for molecular catalysts, which may be easier produced on an industrial relevant scale.

## Conflict of interest

The authors declare no conflict of interest.

1

## Supporting information

As a service to our authors and readers, this journal provides supporting information supplied by the authors. Such materials are peer reviewed and may be re‐organized for online delivery, but are not copy‐edited or typeset. Technical support issues arising from supporting information (other than missing files) should be addressed to the authors.

Supporting Information

## Data Availability

The data that support the findings of this study are available from the corresponding author upon reasonable request.
