# Supplementary material for: Infrared Spectroscopy Elucidates the Inhibitor Binding Sites in a Metal‐Dependent Formate Dehydrogenase
Source: Chemistry. 2022 Aug 3;28(54):e202201091. doi: 10.1002/chem.202201091 (PMC9804402; doi:10.1002/chem.202201091)
Supplement: Supplementary file 1 — Supporting Information [file CHEM-28-0-s001.pdf]

Chemistry–A European Journal

Supporting Information

Infrared Spectroscopy Elucidates the Inhibitor Binding Sites in a Metal-Dependent Formate Dehydrogenase

Konstantin Laun, Benjamin R. Duffus, Stefan Wahlefeld, Sagie Katz, Dennis Belger, Peter Hildebrandt, Maria Andrea Mroginski,* Silke Leimkühler,* and Ingo Zebger*

Materials and Methods

General considerations

The chemicals, reagents, and materials employed in this study were obtained from commercial sources and were of the highest purity. Peptone, yeast extract, plant agar, IPTG, Tris, chloramphenicol, and sodium phosphate were obtained from Duchefa Biochemie (Haarlem, The Netherlands). Ni nitriloacetic acid was obtained from Macherey-Nagel (Düren, Germany). Potassium phosphate was obtained from Merck chemicals (Darmstadt, Germany). Sodium chloride, hydrochloric acid, sodium hydroxide, and potassium hydroxide were obtained from VWR chemicals (Leuven, Belgium). Imidazole, ampicillin, and NaN_3 were obtained from Carl Roth (Karlsruhe, Germany). NaOCN was obtained from Alfa Aesar (Ward Hill, MA). The isotopologues $Na^{15}NN_2$ and $KO^{13}C^{15}N$ were obtained from Eurisotop (Saint-Aubin, France). Sodium formate was obtained from Fluka Biochemika (Steinheim, Germany), while bovine serum albumin (BSA), thionine, DCPIP, and NAD^+ were obtained from Sigma-Aldrich (St. Louis, MO).

Sample preparation

*Rc*FDH^{WT}, *Rc*FDH$^{\Delta bis\text{-}MGD}$, and the respective active site variants *Rc*FDH^{H387M}, and *Rc*FDH^{R587K}, *Rc*FDH$^{H387M/R587K}$ were heterologously expressed in *E.* coli, as described previously.[1] Harvested cells were suspended in lysis buffer (50 mM phosphate, 300 mM NaCl, 10 mM imidazole, pH 8.0) in the presence of 10 mM NaN_3. Subsequent purification steps, including Ni nitrilotriacetic acid-based (Ni-NTA) affinity chromatography and size exclusion chromatography (SEC) using a HiLoad 16/600 Superdex 200 pg column, were performed aerobically in the presence of 10 mM NaN_3 at 4 °C, as was previously performed for the homologously expressed enzyme.[2] The diaphorase subcomplex FdsGB was obtained as a side fraction that accompanied purification of the FdsGBA holoenzyme following SEC. Prior to and immediately after SEC, pooled *Rc*FDH and FdsGB fractions were concentrated with ultracentrifugation devices (Vivaspin 20, 30 kDa MWCO; Sartorius AG, Göttingen, Germany).

Following SEC, *Rc*FDH samples for IR studies were next subjected to Sephadex G-25 desalting by using PD-10 columns (GE Healthcare, Piscataway, NJ) pre-equilibrated with 100 mM Tris-HCl buffer with 10 mM of either NaN_3 or NaOCN (pH 9.0 at 4 °C). Samples containing unlabeled N_3^- were handled aerobically, and were concentrated to ~ 2 mM using Amicon Ultra-0.5 centrifugal filters (100 kDa MWCO) (Merck−Millipore; Billerica, MA) in a cold room. To follow redox changes, sodium formate (10 mM final concentration) was added to the concentrated *Rc*FDH^{WT}, and the sample was flash-frozen for IR measurements; an aliquot was diluted with 100 mM Tris-HCl buffer, 10 mM NaN_3, 10 mM sodium formate (pH 9.0 at 4 °C) and a UV-visible spectrum was obtained on a Shimadzu 2600 spectrophotometer. Sample oxidation with thionine was accomplished in a similar fashion, except that following treatment, the thionine was removed via buffer exchange in Amicon Ultra-0.5 centrifugal filters. *Rc*FDH samples prepared in the presence of $Na^{15}NN_2$ were first made anaerobic via desalting into anoxic 100 mM Tris-HCl, 10 mM NaN_3 (pH 9.0 at 4 °C) in an anaerobic chamber (< 10 ppm O_2; Coy Laboratory Products, Grass Lake, MI); N_3^- exchange was performed by iterative buffer exchange containing 100 mM Tris-HCl, 10 mM $Na^{15}NN_2$ (pH 9.0 at 4 °C) using Amicon Ultra-0.5 centrifugal filters and samples were concentrated using a centrifuge (1−15PK, Sigma, Germany) at 14000g. To minimize sulfido ligand loss, samples containing OCN^- were prepared in a similar fashion anoxically starting with the N_3^--inhibited enzyme, whereby exhaustive removal of N_3^- via PD-10 desalting first into anoxic, inhibitor-free 100 mM Tris-HCl (pH 9.0 at 4 °C) and concentration was performed before desalting into anoxic 100 mM Tris-HCl, 10 mM NaOCN (pH 9.0 at 4 °C). Similar to N_3^-, inhibitor isotopic exchange to $O^{13}C^{15}N^-$ was performed via iterative buffer exchange in an Amicon Ultra-0.5 centrifugal filter into anoxic 100 mM tris-HCl, 10 mM $KO^{13}C^{15}N$ (pH 9.0 at 4 °C).

Following SEC, *Rc*FDH samples for anaerobic inhibition kinetics studies were prepared as described previously for inhibitor-free samples by which the sulfido ligand could be optimally retained.[1c]

Activity measurements

*Rc*FDH activity was characterized by following the oxidation of formate that was coupled to the absorbance changes of using either 2 mM NAD^+ ($\varepsilon_{340\ nm}$ = 6.22 $mM^{-1}cm^{-1}$) or 100 μM 2,6-dichlorophenolindophenol (DCPIP) ($\varepsilon_{600\ nm}$ = 20.7 $mM^{-1}cm^{-1}$) as an electron acceptor, as described elsewhere.[1b, 1c] The effect of N_3^- and OCN^- on the formate oxidation kinetics of RcFDH^{WT}, RcFDH^{H387M} and RcFDH^{R587K} was performed on a Shimadzu 1280 spectrophotometer housed in an anaerobic Coy chamber (O_2 < 10 ppm), working with inhibitor-free enzyme with optimal sulfido ligand content, with sample handling as described elsewhere.[1c] Measurements were performed in 100 mM potassium phosphate buffer at the pH optimum (RcFDH^{WT} pH 9.0, RcFDH^{H387M} pH 7.5, RcFDH^{R587K} pH 8.0). An [N_3^-] of 10 μM – 1 mM, 100 μM – 10 mM, and 1 – 100 mM was employed with 85 nM RcFDH^{WT}, 230 nM RcFDH^{H387M} and 300 nM RcFDH^{R587K}, respectively. Similarly, a [OCN^-] of 250 μM – 20 mM, 1 – 100 mM, and 1 – 100 mM was employed with 85 nM RcFDH^{WT}, 230 nM RcFDH^{H387M} and 300 nM RcFDH^{R587K}, respectively. Measurements were performed at least 5 times from independent protein purifications. Activities were plotted according to the Lineweaver–Burk equation to determine apparent $K_M^{formate}$ values and turnover numbers (k_{cat}) in the presence of excess NAD^+ or DCPIP. k_{cat} values were calculated with respect to one catalytically active protomer $\alpha\beta\gamma\delta$ (MW = 180 kDa). These inhibitor-independent parameters for RcFDH^{WT}, RcFDH^{H387M} and RcFDH^{R587K} in the absence of N_3^- and OCN^- were comparable to previous measurements that had employed NAD^+ as an electron acceptor.[1b] For each combination of *Rc*FDH–inhibitor–electron acceptor, the obtained data was evaluated assuming either competitive or mixed-type inhibition. Where competitive inhibition was observed, the inhibition constant (K_i) was determined using a reciprocal plot of the apparent $K_{m\ app}$ vs. inhibitor concentration. Where mixed-type inhibition (α >1, 0 < β < 1) was observed, the respective K_i and αK_i values were determined by plotting the $K_{m\ app}$/v and 1/v vs. inhibitor concentration via linear regression.[9] Comparable values were obtained upon treatment of the obtained data using a non-linear fit. All analyses and graphing were performed in OriginPro 2021 (OriginLab Corporation, Northampton, MA). NAD^+ and DCPIP were compared as electron acceptors to assess whether differences in inhibition might be due to inhibition of the redox relay; NAD^+ accepts electrons from the enzyme from the diaphorase subcomplex.

Metal determination

*Rc*FDH % Mo and % Fe saturations were determined via inductively coupled plasma–optical emission spectroscopy (ICP–OES), as described elsewhere.[1a] Saturations were calculated based relative to complete saturation of the bis-MGD and the 7 Fe-S clusters predicted per protomer. *Rc*FDH^{WT}, *Rc*FDH^{H387M}, *Rc*FDH^{R587K}, *Rc*FDH$^{H387M/R587T}$ and *Rc*FDH$^{\Delta FdsC}$ had % Mo and % Fe saturations of 49.9 ± 2.3 % and 49.1 ± 1.8 %, 45.6 ± 2.9 % and 52.6 ± 0.8 %, and 46.3 ± 2.5 % and 52.1 ± 1.3 %, 14.6 ± 0.8 % and 45.4 ± 0.9 %, and 39.7 ± 1.1 % and 53.8 ± 1.4 % respectively. The FdsGB subcomplex used for IR measurements had a % Fe saturation of 47.4 ± 0.3 %. The bis-MGD lacking apoenzymes *Rc*FDH$^{\Delta bis\text{-}MGD}$, *Rc*FDH$^{\Delta bis\text{-}MGD(H387M)}$, and *Rc*FDH$^{\Delta bis\text{-}MGD(R587K)}$ had % Fe saturations of 53.2 ± 1.2 %, 53.1 ± 1.5 %, and 56.0 ± 1.7 % respectively.

Density functional theory (DFT) based calculation

In order to investigate the binding of a N_3^- or an OCN^- anion to the bis-MGD cofactor, six structural models were generated, optimized and submitted to vibrational frequency calculations based on Density Functional Theory (DFT). These models were built using as template the atomic arrangement of the bis-MGD found in the crystal structure of oxidized FDH from *E. coli* (PDB entry: 1KQF).[3] For simplification, a truncated model of the bis-MGD cofactor was considered which included the dithiolene ligands coordinated to the Mo metal, as well as the two pterin moieties. All models involve a hexacoordinated Mo^{VI} with an apical S2-ligand and the sulphur of a Cys at the sixth coordination site. The cysteine amino acid is cut at its C_α position and valencies are saturated with hydrogen atoms. Two possible binding modes of each inhibitor to the bis-MGD cofactor were considered: the inhibitor covalently binds to the Mo at the cysteine coordination site, replacing the Cys and, the inhibitor is placed in the vicinity of the bis-MGD cofactor without forming any chemical bond (Figure S4). All quantum chemical calculations were performed with the Gaussian16 suite of programs,[4] using the BP86 functional[5] combined with the 6-31G* basis set for H, O, N and the def2-TVPP basis sets for S and Mo.[6] Additionally, energy adjusted *ab-initio* pseudopotentials for the Mo core was employed in order to reduce computational cost and simultaneously improve the description of relativistic effects.[7] The final optimized structures are shown in Fig. S4.

Vibrational frequencies and normal modes of vibrations were computed using the normal mode analysis approach implemented in Gaussian16. IR intensities were estimated from the spatial derivatives of the electric dipole moments and the corresponding IR spectra were plotted assuming Lorentzian band shapes with bandwidths of 12 cm^{-1}.

The protein environment has been mimicked in all structural models through polarizable continuum model with a dielectric field constant of ε=4.[8] For comparison, geometry optimization and frequency calculation of N_3^- and OCN^- molecule in water were perform at the same level of theory as employed for the models described above. In this case, the solvent was modeled through a polarizable continuum model with a dielectric constant of constant of ε = 80.

With the help of DFT calculations, we can exclusively predict whether the azide or isocyanate molecules covalently bind to the Mo ion, or are simply non-covalently interacting with the bis-MGD cofactor. The shifts of the N_3^-/OCN^- stretching frequencies upon covalent binding are predicted to be upshifted more than 50 cm^{-1} (see Fig 3 and Fig. S6), while the stretching frequencies of inhibitors, which are not covalently bound to the Mo ion, are in excellent agreement with the experimental IR peaks (2031 and 2153 cm^{-1} for N_3^- and NCO, respectively). Thus, these DFT calculations simply refute a covalent binding of the inhibitor to the Mo ion. The exact binding site of the inhibitor in the bis-MGD binding pocket or even in the protein, however, cannot be predicted by these DFT calculations. The fact that the vibrational frequency bands at 2031 and 2153 cm^{-1} are only detected when the bis-MGD cofactor is present (Fig.2) indicates that the inhibitor molecules are directly or indirectly interacting with it, and the high analogy of N_3^- and NCO^- to CO_2 would suggest a similar binding site for the two inhibitors, namely somewhere in the vicinity of the Mo ion. Therefore, based on these assumptions, the bis-MGD models with non-covalently interacting N_3^-/OCN^- were constructed by placing these molecules in the vicinity of the bis-MGD and optimizing their geometry towards a *local energy minimum*. The absence of covalent interaction with the Mo ion is confirmed by estimating the shortest distances between inhibitor and Mo ion (see Table S1). According to the predicted distances of the inhibitors to the Mo ion, it was shown that azide and cyanate are rather orientated towards the sulfido ligand than to the Mo ion itself. This could explain the experimentally observed shift of the bis-MGD dependent band at 2031 cm^{-1} which shifts to 2034 cm^{-1} when the sulfide is exchanged to an oxo ligand (Mo=O in the *Rc*FDH$^{\Delta FdsC}$), see Fig. S5. In addition, calculation of the vibrational properties of the free ions in

solution (modeled via a polarizable continuum model) and comparison with the vibrational signature of ions in the vicinity of the bis-MGD, yield the same trend as observed experimentally (see Fig. 3). These calculations confirm the experimental assignment and probe the accuracy of the theoretical approach.

Table S1. Predicted shortest distances of the inhibitory anions at the bis-MGD cofactor by reaching a *local energy minimum*.

interaction mode	interaction distance (Å)
N_3^-....Mo	3.9
N_3^-....S=Mo	2.42
NCO^-....Mo	5.7
OCN^-....Mo	5.11
NCO^-....S=Mo	3.785
OCN^-....S=Mo	3.294

Infrared Spectroscopy

For infrared (IR) transmission measurements, a Bruker Vertex 70v Fourier Transformation IR spectrometer equipped with a liquid N_2 mercury cadmium telluride (MCT) detector was used. The IR transmission spectra were recorded with a spectral resolution of 2 cm^{-1}. *Rc*FDH samples were measured in a temperature-controlled (10°C), gas-tight IR transmission cell (CaF_2 windows, volume = 6 – 10 μL, optical path length = 50 μm) placed in the spectrometer's sample compartment, which was purged with dried air.

For single channel spectra, 200 scans were accumulated. An average of 10 single channel spectra of the sample and the background was then used to obtain absorbance spectra by using the Beer-Lambert law (see Fig. S1). The further spectra analysis, such as the calculation of difference spectra, as well as baseline corrections, was performed using the OPUS 7.8 software (Bruker). The figure plots were created with OriginPro 2021 (OriginLab Corporation, Northampton, MA).

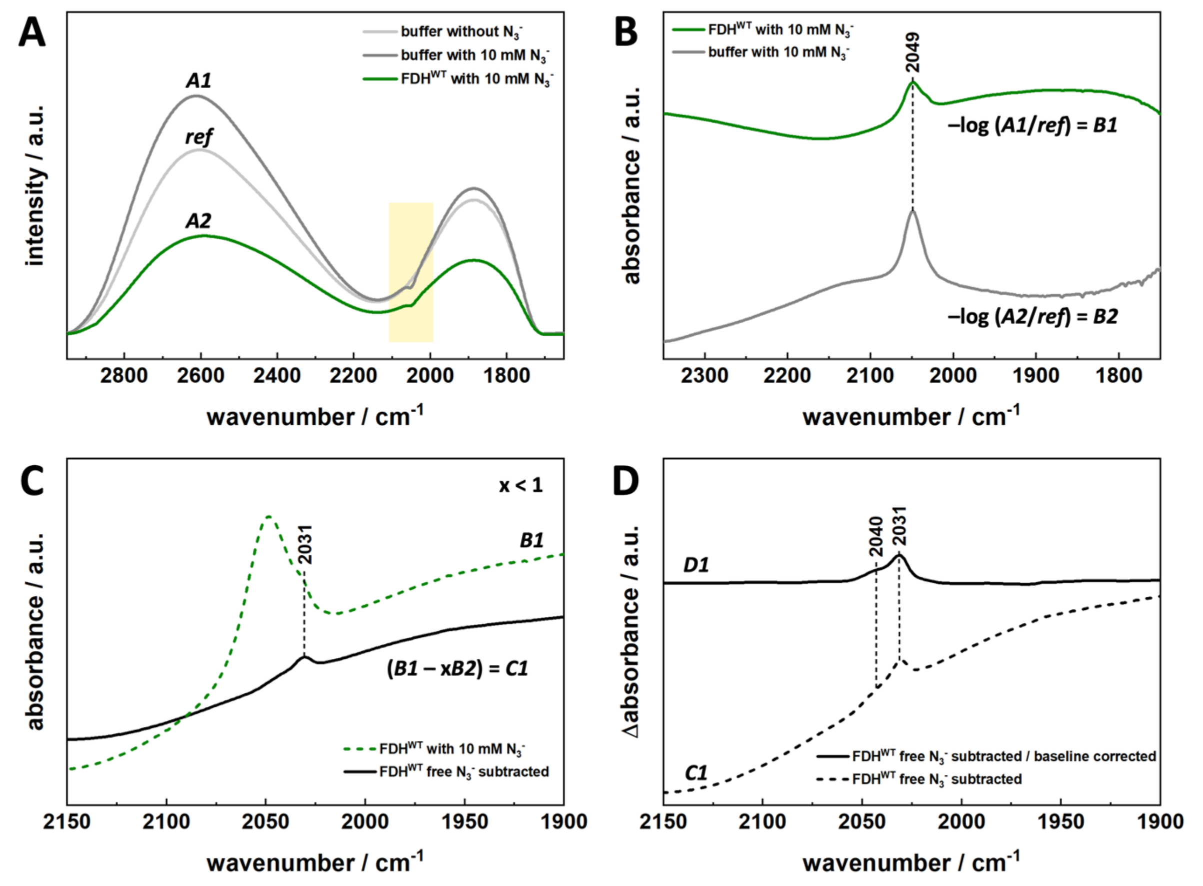

Figure S1. Procedure for IR spectroscopic data evaluation. For each measurement, the corresponding spectra were processed stepwise: In panel (A) averaged single channel spectra are displayed for the bare buffer (Tris-HCl 50 mM, pH 9) as trace "*ref*", the buffer with N_3^- (50 mM tris-HCl, 10 mM NaN_3, pH 9) as trace *A1* and the protein solution (*Rc*FDH^{WT}, 50 mM tris-HCl, 10 mM NaN_3, pH 9) as trace *A2*. These spectra were used, as displayed in panel (B), to calculate the corresponding absorbance spectra via Beer-Lamberts law for dissolved N_3^- in the buffer and the enzyme solution incubated with N_3^-, using the bare buffer as reference / background. The resulting absorbance spectra *B1* and *B2* are both dominated by a main peak located at 2049 cm^{-1}, which refers to the free N_3^- dissolved in buffer (*B2*). To remove its spectral contribution from the *B1* spectrum of the N_3^- incubated enzyme, the corresponding *B2* spectrum was firstly multiplied with a factor in order to reach equal band intensities and subsequently subtracted from the *B1* spectrum resulting in a difference spectrum *C1* excluding contributions of N_3^- absorption, see panel (C). Afterwards, the corresponding spectrum *C1*

was manually baseline corrected (D1 in panel D). The origin of the remaining bands at 2031 and 2040 cm^{-1} is discussed in detail in the manuscript. The studied *Rc*FDH samples were concentrated to 1 – 2 mM with respect to the Mo loading. Thus, spectra shown in the manuscript are generally normalized to protein concentration.

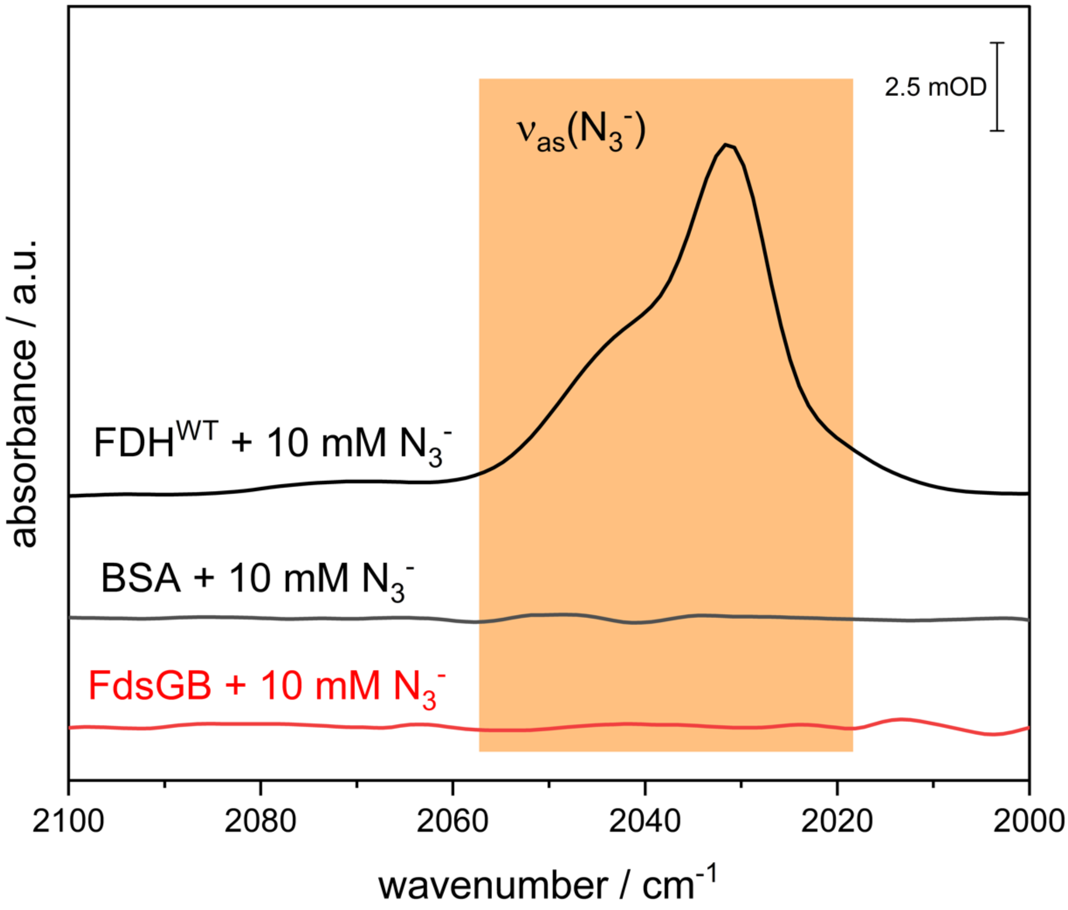

Figure S2. IR spectroscopic investigation of potential unspecific azide binding to the protein backbone. Comparison of IR absorbance spectra of *Rc*FDH^{WT}, Bovine serum albumin (BSA, lyophilized powder diluted in 100 mM KPO_4 buffer, pH 7 and 10 mM azide) and FdsGB, a *Rc*FDH variant that lacks the bis-MGD harboring α -subunit (pH 9, 100 mM Tris buffer and 10 mM azide). The spectra of these control experiments, after subtraction of free azide, show unambiguously, that no unspecific binding in the protein backbone of BSA or FdsGB occurs. In addition, the absence of the α-subunit in the *Rc*FDH protein results also in a loss of the corresponding band representing the competitive binding at lower frequencies, suggesting, that both binding sites are present in the FdsA subunit. IR spectra were evaluated by the procedure described in Figure S1.

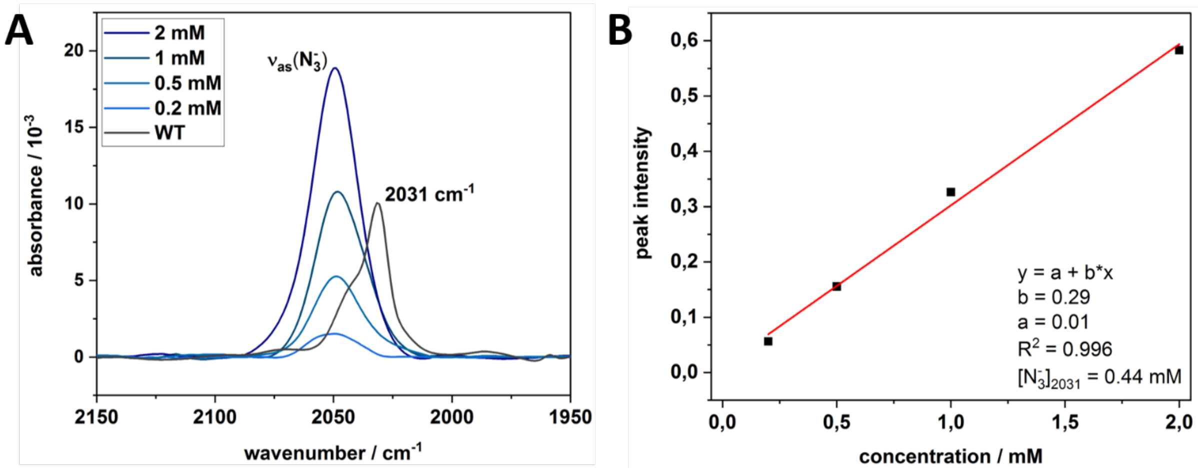

Figure S3. IR spectroscopic quantitation of the amount of inhibiting N_3^- species bound in *Rc*FDH^{WT} using a dilution series of N_3^- dissolved in pure buffer as reference (A). Herein, spectra of aliquots with different amounts of N_3^- (0.2 – 2.0 mM) dissolved in 100 mM Tris-HCl buffer at pH 9.0 were recorded in an IR transmission cell. The integrated absorbance/intensity of the antisymmetric stretching vibration (ν_{freeN3} = 2049 cm^{-1}) is plotted as a function of the azide concentration, as shown in (B). As a similar extinction coefficient for free and bound N_3^- cannot be assumed *per se*, the formula displayed in panel B can be therefore used only in a semi-quantitative approximation. In such way, for *Rc*FDH^{WT} an inhibitory N_3^- concentration of 0.44 mM was estimated for the observed IR absorption band at 2031 cm^{-1}. The band intensity at 2040 cm^{-1} was estimated via the same semi-quantitative approach to be ca. 0.25 mM. The sample contained 1.4 mM *Rc*FDH^{WT} with ~ 50 % occupancy of the bis-MGD cofactor. For *Rc*FDH^{WT} and *Rc*FDH$^{\Delta bis\text{-}MGD}$ as well as for all other measured variants, ~ 0.5 N_3^- anions per *Rc*FDH protein were assessed. It is important to emphasize here, that it is not clear how the particular binding mode in *Rc*FDH affects the extinction coefficient and, thus, the height of related IR absorption. Moreover, the experimentally derived band intensity is also affected by the subtraction procedure of free N_3^- as well as by the baseline correction. Hence, the herein calculated values can be considered only as an approximation. From this data, we tentatively infer one bis-MGD-dependent and one bis-MGD-independent binding site harboring an azide molecule in a stoichiometric manner for *Rc*FDH–N_3 interaction. After the analysis of several cyanate inhibited *Rc*FDH samples, it turned out that our semi-quantitative approximation is highly error-prone for a weak inhibitor such as cyanate, due to its relatively low extinction coefficient. Therefore, we decided to analyze only the characteristic absorption bands of the azide inhibited samples with our approach.

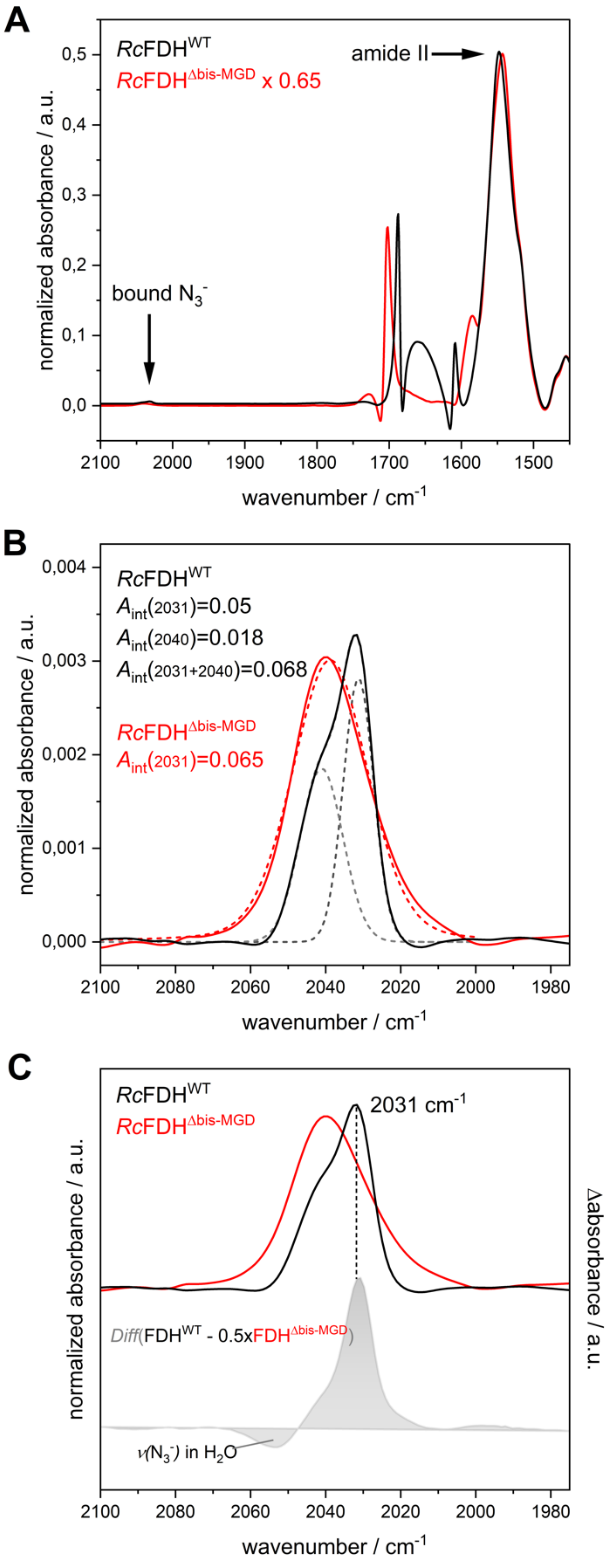

Figure S4. IR spectroscopic comparison of azide binding in holo- and apoenzyme of *RC*FDH. IR absorbance spectra were generated by subtraction of the free azide in water (ν_{freeN3} = 2049 cm^{-1}), see Fig S1, and normalized to the amide II band. The baseline correction was conducted after normalization. Herein, the IR spectrum of the *Rc*FDH^{WT} (black) was chosen as a reference, while the *Rc*FDH$^{\Delta bis\text{-}MGD}$ spectrum (red) had to be multiplied with a factor of 0.65 to reach the

same amide II intensity (A). Subsequently, the normalized spectra were analyzed in the frequency regime of the azide stretching vibrations between 2000 – 2100 cm^{-1}. The integral absorbance (A_{int}) of bound azide species was derived by a band fit. For *Rc*FDH^{WT}, the corresponding IR absorptions for competitive and the non-competitive bound azide species at 2031 and 2040 cm^{-1} yielded an overall integral absorbance value of A_{int} = 0.068. Contrarily, the *Rc*FDH$^{\Delta bis\text{-}MGD}$ enzyme exhibits only a non-competitive azide binding site, with an estimated integral absorbance of A_{int} = 0.065 (B). This finding supports the hypothesis that the azide molecule can interact with different binding motifs within in the protein backbone dependent on the presence of the bis-MGD cofactor. In order to elucidate such interaction sites, the spectral contribution of the unloaded *Rc*FDH (2040 cm^{-1}) can be eliminated from the spectrum of the holoenzyme by subtracting the *Rc*FDH$^{\Delta bis\text{-}MGD}$ data multiplied by a factor of 0.5, considering a bis-MGD cofactor occupancy of 50% (0.5 Mo/FDH). This subtraction yields the corresponding IR difference spectrum *Diff*(*Rc*FDH^{WT} – 0.5x *Rc*FDH$^{\Delta bis\text{-}MGD}$) with a predominant absorption at 2031 cm^{-1} as shown in (C). This clearly confirms two distinct binding motifs for azide in the secondary coordination sphere, one of which is dependent on the bis-MGD cofactor exhibiting a competitive binding site (2031 cm^{-1}). In addition, also the band shape and the relative band/frequency shifts of both binding sites provide further information on the particular interaction. The bis-MGD dependent band at 2031 cm^{-1} exhibits a more Lorentzian-like shape band profile with a smaller band width, due to its more distinct interactions with the bis-MGD and amino acid residues, which is most likely the Arg587 and in particular the sulfido ligand. In the absence of the Arg587 or the bis-MGD cofactor, a more flexible binding motif is formed in which the number of degrees of translational freedom potentially increases, which results in a broader absorption band at 2040 cm^{-1}. This assumption is supported by the observed band shift towards the stretching frequency of free azide in water (ν_{freeN3} = 2049 cm^{-1}).

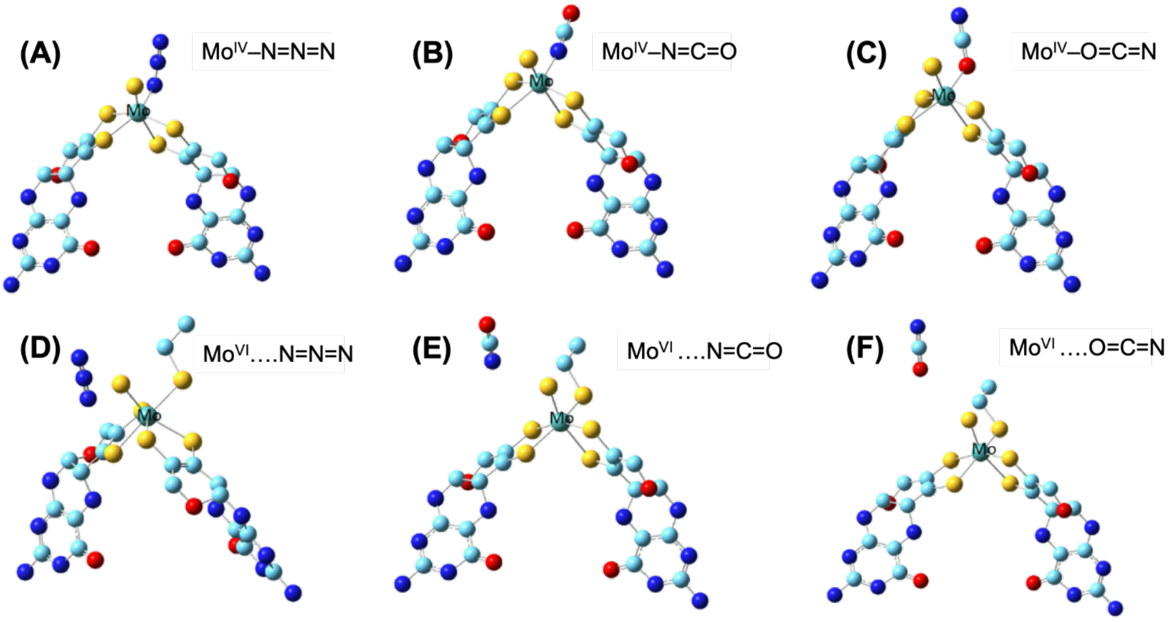

Figure S5. Optimized geometries of the bis-MGD cofactor models inhibited with N_3^- (A and D) and OCN^- (B, C, E, F), considering covalent binding (top) and non-covalent binding (bottom) arrangements. For clarity, hydrogen atoms are not depicted. Except for the Cys386, no further amino acid residues are included into the optimized model. For non-bonded inhibitors, the respective inhibitory anion is kept in a stable position near the Mo center. Geometries in A and D are presented with the corresponding experimental IR data in the manuscript in Fig. 3. The geometries in B and E, shown with corresponding IR data in Fig. S7, are calculated using an OCN^- anion with either a Mo-N bond or an orientation of the terminal nitrogen towards the Mo ion, where the OCN^- anion is kept stable near the bis-MGD cofactor. The scenario in C and F exhibits an orientation of the oxygen (Mo–O or Mo…O) towards the bis-MGD cofactor (see Fig. 3 in manuscript).

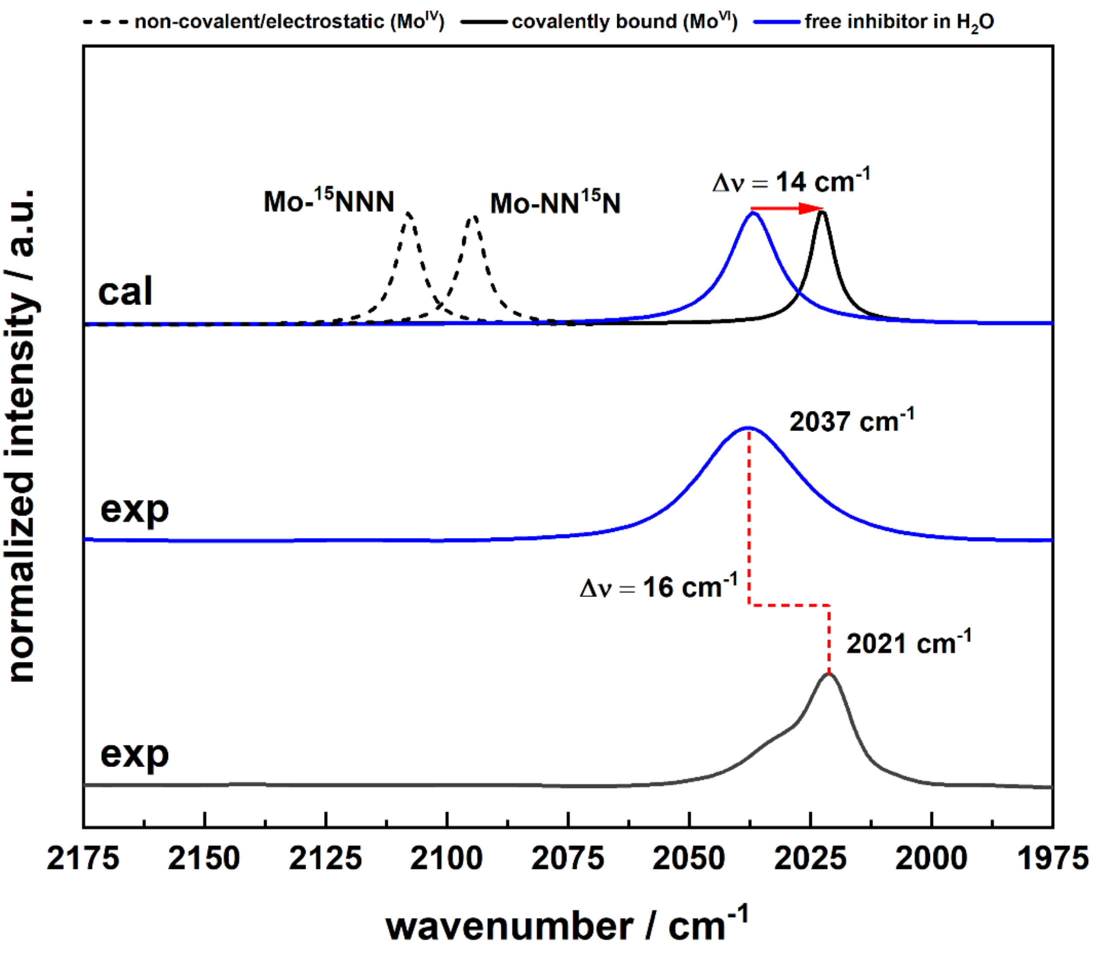

Figure S6. Experimentally recorded IR spectra of *Rc*FDH^{WT} inhibited with terminally isotopic-labeled ^{15}N azide ($N{=}N{=}^{15}N^-$) anion depicted together with the corresponding calculated spectra. For the DFT calculations and the experiments in water (buffer), an isotropic orientation of the terminally labeled anion within the medium was assumed. The corresponding calculated stretching vibrational modes were derived for $N{=}N{=}^{15}N^-$ which is bound covalently either via the ^{14}N or the ^{15}N to the Mo ion (dashed trace, Mo–$N{=}N{=}^{15}N$/$^{15}N{=}N{=}N$) or alternatively, kept in an energetically favored position near the sulfido ligand at the Mo ion (solid black trace) and are shown in the top layer. As a reference, $N{=}N{=}^{15}N^-$ in water with an isotropic orientation is shown in dark blue. The corresponding absorption of $N{=}N{=}^{15}N^-$ in buffer is shown in the middle layer as blue trace. Generally, the calculations predict, relative to free N_3^- in water, a red shift ($\Delta\nu_{cal}$ = -14 cm^{-1}) for non-covalent interaction with the bis-MGD cofactor. A blue shift towards higher wavenumbers is calculated for a covalent/coordinate bond of $N{=}N{=}^{15}N^-$ to the Mo ion. This was also described by Leone and co-workers for a Cu/Zn superoxide dismutase that binds N_3^- only in a reduced state and not in

the oxidized state.[10] Experimentally, for $N=N=^{15}N^{-}$ in *Rc*FDH^{WT} a red shift of $\Delta\nu_{exp}$ = -16 cm^{-1} relative to the free $N=N=^{15}N^{-}$ in water (ν = 2037 cm^{-1}) is observed, in line with electrostatic interaction with the bis-MGD cofactor. Furthermore, none of the terminal atoms (^{14}N /^{15}N) from the $N=N=^{15}N^{-}$ anion seem to be favored to form a covalent bond with the Mo ion in regard of a specific orientation. Thus, both species (Mo–N=N=^{15}N / Mo–^{15}N=N=N) could be formed. This observation was described by Yoshikawa and co-workers for a cytochrome c oxidase[11] and was also demonstrated by the related DFT calculations. In principle, the same scenario could be considered for a labeled/non-labeled OCN^{-} anion, but compared to the N_3^{-} species, a favored orientation via an N or O atom (Mo–N=C=O or Mo–O=C=N) cannot be excluded. Notably, no additional band was observed experimentally.

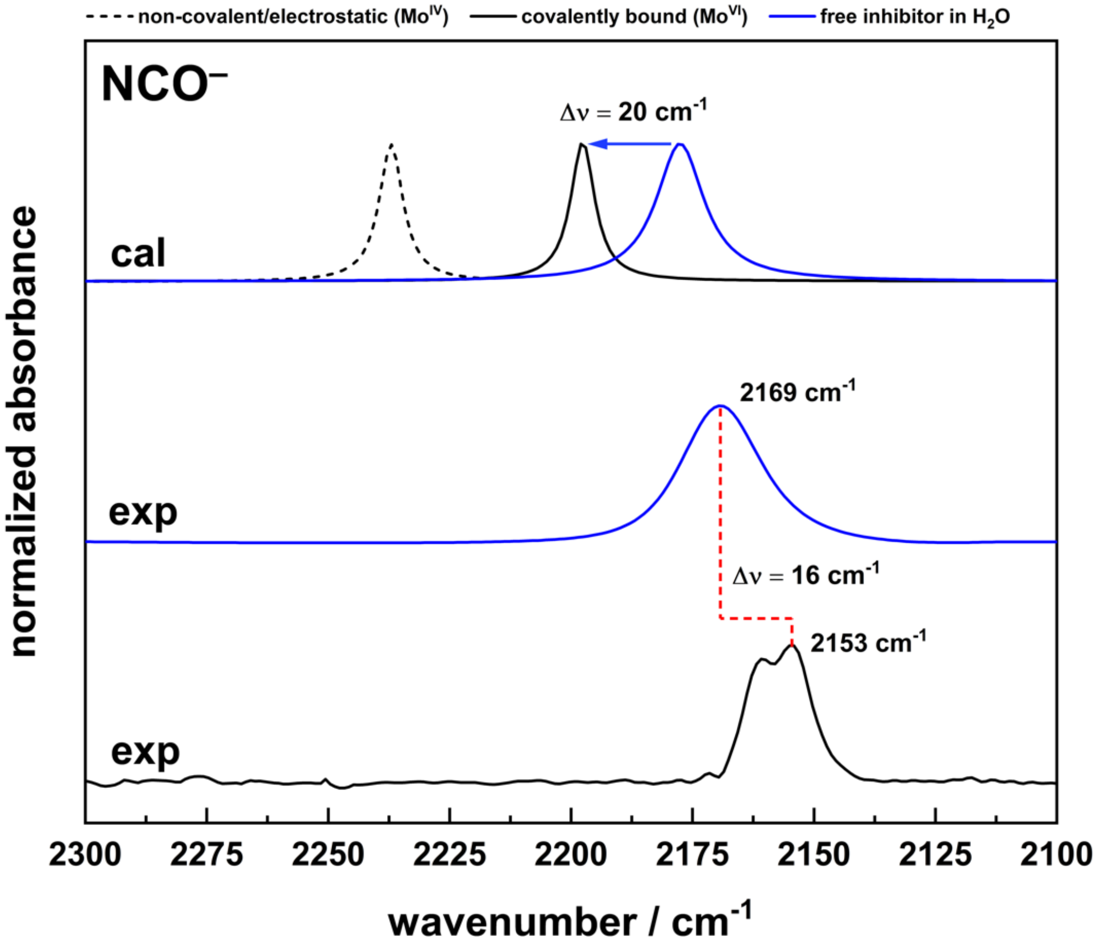

Figure S7. Calculated IR spectra of cyanate (NCO^-) stretching vibrations for an alternative orientation (see Fig. S5) relative to the bis-MGD cofactor (top). Hereby, the corresponding absorption frequencies for free cyanate in water (dark blue), a coordinate Mo—N bond (dashed line) or inverted alignment of NCO^- (black line) for a non-covalent interaction were calculated. Notably, a blue shift was derived from calculations of both coordinate bond and non-covalent interaction. However, for the latter, a much smaller shift of $\Delta\nu_{cal}$ = +20 cm^{-1} with respect to free NCO^- was predicted. Experimentally, a red shift of $\Delta\nu_{exp}$ = –16 cm^{-1} is observed (bottom, black) in comparison to the band position of free NCO^- in buffer (middle, blue trace). This might be an indication for a preferential electrostatic inhibition for cyanate with an orientation, in which the oxygen atom is aligned towards the bis-MGD cofactor (see Fig. 3 in the manuscript). However, additional interaction with amino acid residues near the cofactor may also have an impact on the exact position of the respective IR absorption band.

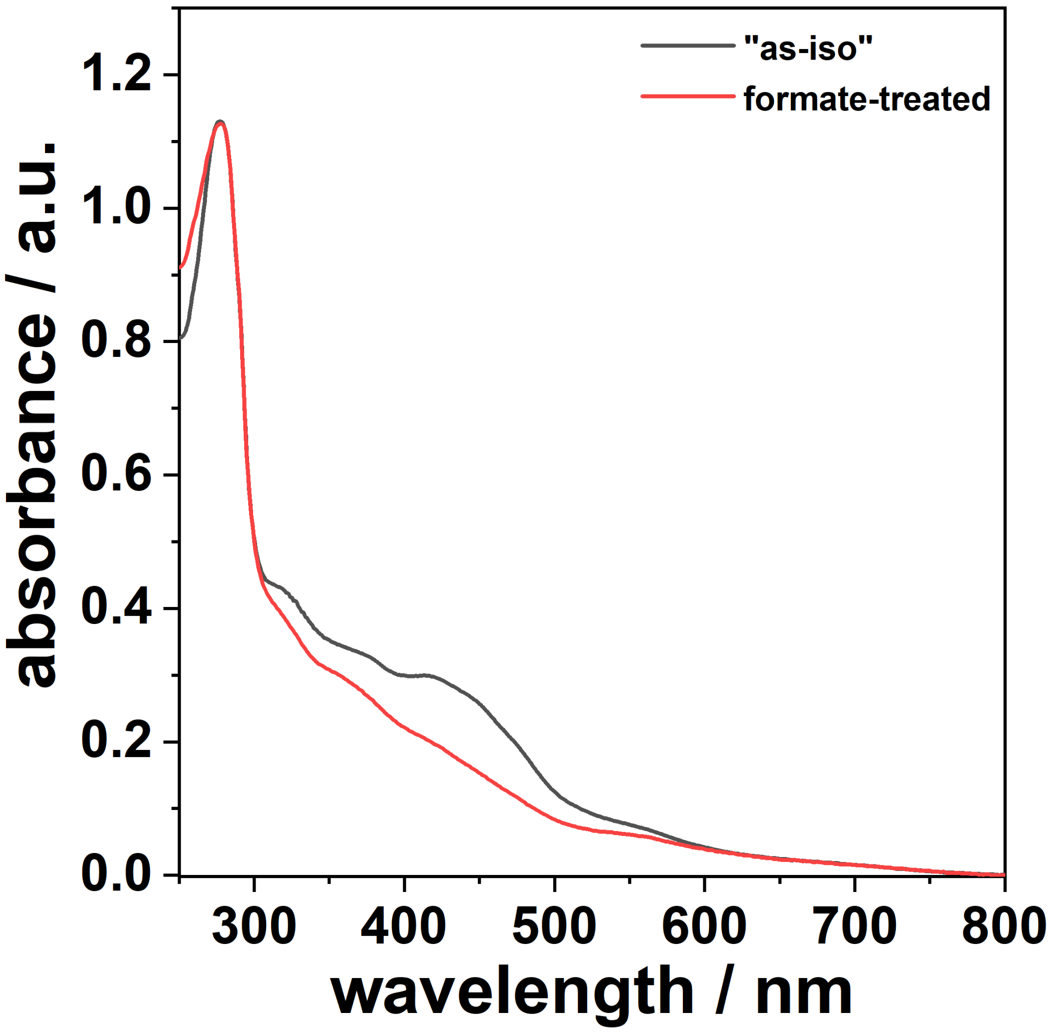

Figure S8. UV-visible spectra of *Rc*FDH^{WT} IR samples in the "as isolated" and reduced form in presence of N_3^-. Herein, the displayed UV-visible spectra were recorded on the same with N_3^- incubated protein samples used also in the corresponding IR measurements, as depicted in Fig. 2. Spectra were recorded of *Rc*FDH^{WT} in 100 mM Tris-HCl with 10 mM NaN_3 at pH 9.0 and 4 °C in the presence or absence of 10 mM sodium formate. The reduction of the active site and the Fe-S clusters was confirmed by the pronounced intensity loss in the absorbance regime between 300 and 500 nm.

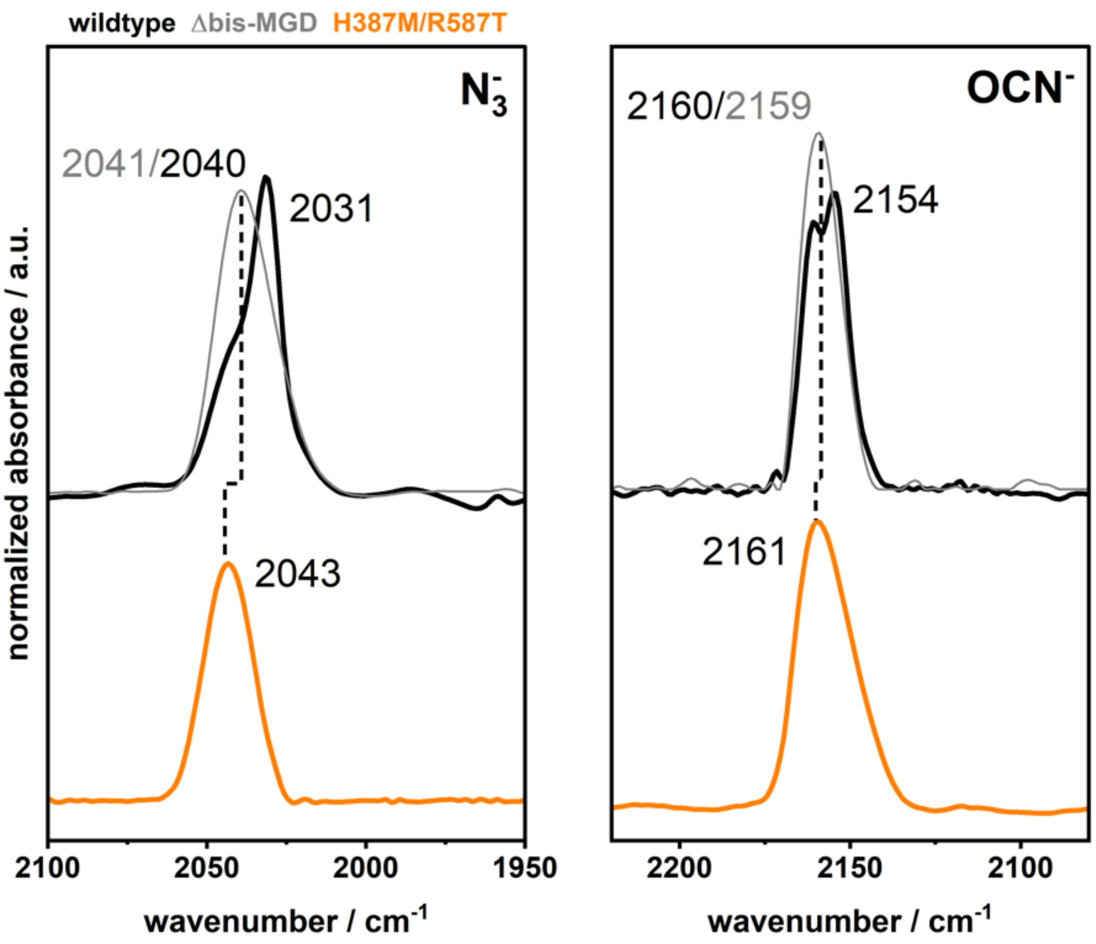

Figure S9 IR spectroscopic data derived from the double variant $FDH^{H387M/R587K}$ (orange traces) after incubation with azide and cyanate, respectively. The high frequency band, representing non-competitive inhibition (see manuscript), exhibits a shift towards higher frequencies upon substitution of the amino acid residues H387 and R587. Since the corresponding low frequency absorption observed at 2031 cm^{-1} for azide and at 2054 cm^{-1} for cyanate disappear in the absence of these two conserved amino acid residues, it can be assumed that they form the competitive binding site near the bis-MGD, but not the second, non-competitive binding site that is more remote. However, they might nevertheless have an effect on the non-competitive binding site.

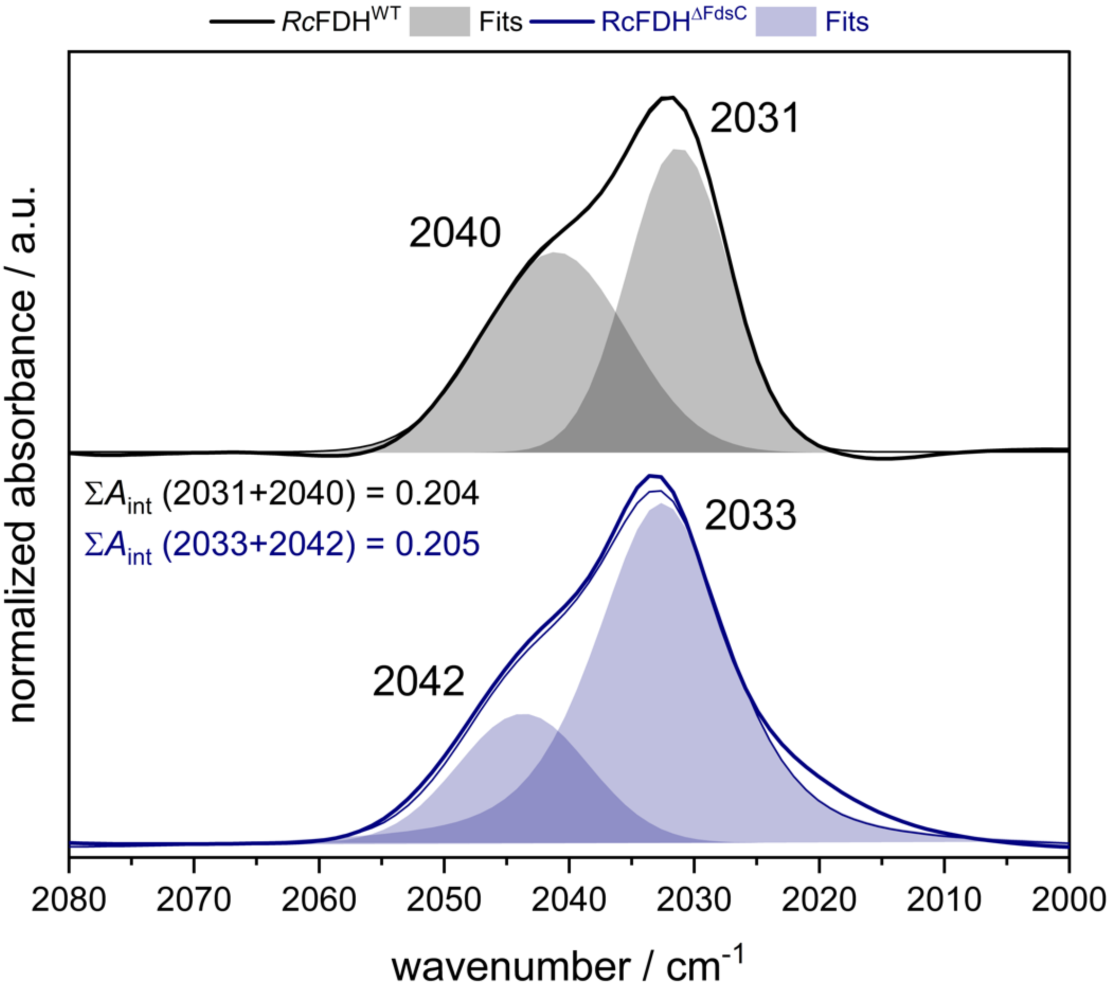

Figure S10. Semi-quantitative band/component evaluation of the *Rc*FDH^{WT} (upper black trace) and *Rc*FDH$^{\Delta FdsC}$ (upper dark blue trace) spectra. The absorption of the competitive binding azide species is upshifted from 2031 to 2033 cm^{-1}. However, the ratio of both azide species seems to be slightly changed. Herein, in the *Rc*FDH$^{\Delta FdsC}$ spectrum the relative band intensity at 2033 cm^{-1} clearly increases compared the band at 2042 cm^{-1} by contrast with the *Rc*FDH^{WT} spectral data. Nevertheless, the overall integral absorbance A_{int} remains about constant during the inactivation of the bis-MGD cofactor. Both samples were normalized to the respective amide II band intensity, as illustrated in Fig. S4. In order to ensure, that the absorbance of amide II band remains in the linear regime, both samples were measured in an IR transmission cell implementing a 20 µm spacer.

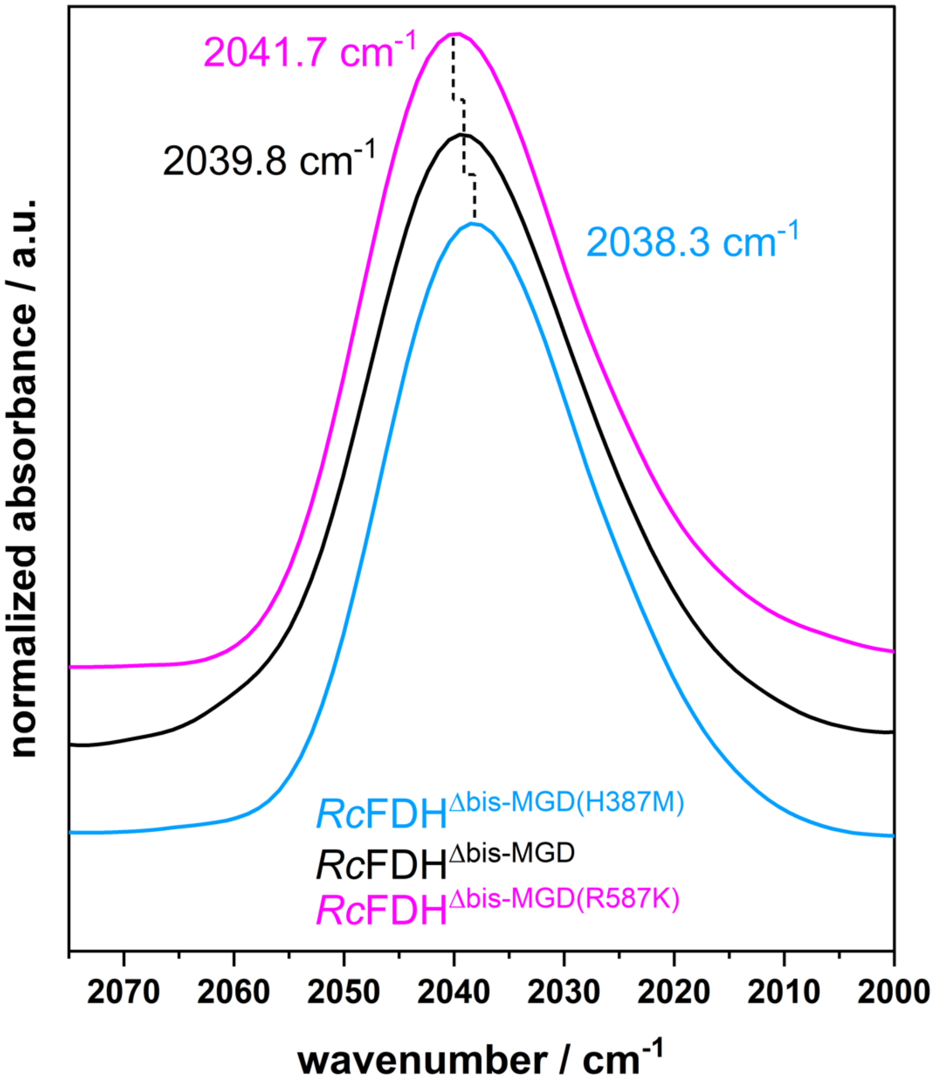

Figure S11. Normalized IR spectra of apo protein variants. Comparison of *Rc*FDH protein *Rc*FDH$^{\Delta bis\text{-}MGD}$, and the active site variants *Rc*FDH$^{\Delta bis\text{-}MGD(R587K)}$ and *Rc*FDH$^{\Delta bis\text{-}MGD(H387M)}$, all of which without embedded bis-MGD. The spectrum of holo-*Rc*FDH^{WT} reveals two bands related to azide interaction with the protein, one of which is also present in the *Rc*FDH$^{\Delta bis\text{-}MGD}$. The corresponding band position of this non-competitive inhibition site occurs at 2039.8 cm^{-1}. For the *Rc*FDH$^{\Delta bis\text{-}MGD(R587K)}$, a frequency upshift to 2041.7 cm^{-1} was observed. In contrast, the *Rc*FDH$^{\Delta bis\text{-}MGD(H387M)}$ shifts towards lower frequencies (2038.3 cm^{-1}) in the absence of the bis-MGD. Small spectral shifts of the absolute band positions relative to the frequencies observed in the respective holo enzymes are presumably related could be explained by the laborious and error-prone procedure for the baseline correction and subtraction of the free azide absorption in water.

References

[1] a) T. Hartmann, S. Leimkühler, *FEBS J.* 2013, *280*, 6083-6096; bT. Hartmann, P. Schrapers, T. Utesch, M. Nimtz, Y. Rippers, H. Dau, M. A. Mroginski, M. Haumann, S. Leimkühler, *Biochemistry* 2016, *55*, 2381-2389; b) B. R. Duffus, P. Schrapers, N. Schuth, S. Mebs, H. Dau, S. Leimkühler, M. Haumann, *Inorg. Chem.* 2020, *59*, 214-225.

[2] C. Radon, G. Mittelstadt, B. R. Duffus, J. Burger, T. Hartmann, T. Mielke, C. Teutloff, S. Leimkühler, P. Wendler, *Nat. Commun.* 2020, *11*, 1912.

[3] M. Jormakka, S. Tornroth, B. Byrne, S. Iwata, *Science* 2002, *295*, 1863-1868.

[4] M. J. Frisch, G. W. Trucks, H. B. Schlegel, G. E. Scuseria, M. A. Robb, J. R. Cheeseman, G. Scalmani, V. Barone, G. A. Petersson, H. Nakatsuji, X. Li, M. Caricato, A. V. Marenich, J. Bloino, B. G. Janesko, R. Gomperts, B. Mennucci, H. P. Hratchian, J. V. Ortiz, A. F. Izmaylov, J. L. Sonnenberg, Williams, F. Ding, F. Lipparini, F. Egidi, J. Goings, B. Peng, A. Petrone, T. Henderson, D. Ranasinghe, V. G. Zakrzewski, J. Gao, N. Rega, G. Zheng, W. Liang, M. Hada, M. Ehara, K. Toyota, R. Fukuda, J. Hasegawa, M. Ishida, T. Nakajima, Y. Honda, O. Kitao, H. Nakai, T. Vreven, K. Throssell, J. A. Montgomery Jr., J. E. Peralta, F. Ogliaro, M. J. Bearpark, J. J. Heyd, E. N. Brothers, K. N. Kudin, V. N. Staroverov, T. A. Keith, R. Kobayashi, J. Normand, K. Raghavachari, A. P. Rendell, J. C. Burant, S. S. Iyengar, J. Tomasi, M. Cossi, J. M. Millam, M. Klene, C. Adamo, R. Cammi, J. W. Ochterski, R. L. Martin, K. Morokuma, O. Farkas, J. B. Foresman, D. J. Fox, Wallingford, CT, 2016.

[5] A. D. Becke, *Phys. Rev. A* 1988, *38*, 3098-3100.

[6] F. Weigend, R. Ahlrichs, *Phys. Chem. Chem. Phys.* 2005, *7*, 3297-3305.

[7] D. Andrae, U. Haussermann, M. Dolg, H. Stoll, H. Preuss, *Theor. Chim. Acta* 1990, *77*, 123-141.

[8] J. Tomasi, B. Mennucci, R. Cammi, *Chem. Rev.* 2005, *105*, 2999-3093.

[9] I. H. Segel, *Enzyme Kinetics: Behavior and Analysis of Rapid Equilibrium and Steady-State Enzyme Systems*, Wiley-Interscience, Hoboken, NJ, 1975.

[10] M. Leone, A. Cupane, V. Militello, M. E. Stroppolo, A. Desideri, *Biochemistry* 1998, *37*, 4459-4464.

[11] S. Yoshikawa, W. S. Caughey, *J. Biol. Chem.* 1992, *267*, 9757-9766.
